# Airborne transmission pathway for coastal water pollution

**DOI:** 10.7717/peerj.11358

**Published:** 2021-06-07

**Authors:** Matthew A. Pendergraft, Derek J. Grimes, Sarah N. Giddings, Falk Feddersen, Charlotte M. Beall, Christopher Lee, Mitchell V. Santander, Kimberly A. Prather

**Affiliations:** 1 Scripps Institution of Oceanography, University of California San Diego, La Jolla, California, United States of America; 2 Department of Chemistry and Biochemistry, University of California San Diego, La Jolla, California, United States

**Keywords:** Coastal pollution, Air pollution, Sea spray aerosol, Water pollution, Water quality, Tijuana River, Tracer dye, Imperial Beach, Surfzone, Aerosols

## Abstract

Each year, over one hundred million people become ill and tens of thousands die from exposure to viruses and bacteria from sewage transported to the ocean by rivers, estuaries, stormwater, and other coastal discharges. Water activities and seafood consumption have been emphasized as the major exposure pathways to coastal water pollution. In contrast, relatively little is known about the potential for airborne exposure to pollutants and pathogens from contaminated seawater. The Cross Surfzone/Inner-shelf Dye Exchange (CSIDE) study was a large-scale experiment designed to investigate the transport pathways of water pollution along the coast by releasing dye into the surfzone in Imperial Beach, CA. Additionally, we leveraged this ocean-focused study to investigate potential airborne transmission of coastal water pollution by collecting complementary air samples along the coast and inland. Aerial measurements tracked sea surface dye concentrations along 5+ km of coast at 2 m × 2 m resolution. Dye was detected in the air over land for the first 2 days during two of the three dye releases, as far as 668 m inland and 720 m downwind of the ocean. These coordinated water/air measurements, comparing dye concentrations in the air and upwind source waters, provide insights into the factors that lead to the water-to-air transfer of pollutants. These findings show that coastal water pollution can reach people through an airborne pathway and this needs to be taken into account when assessing the full impact of coastal ocean pollution on public health. This study sets the stage for further studies to determine the details and importance of airborne exposure to sewage-based pathogens and toxins in order to fully assess the impact of coastal pollution on public health.

## Introduction

Roughly half of the global population lives in coastal regions (U.S. Comission on Ocean Policy, 2004). The discharge of treated and untreated sewage, industrial effluents, agricultural drainage, and urban stormwater into coastal waters is a global public health concern (U.S. Comission on Ocean Policy, 2004; Shuval, 2003; Halpern et al., 2012). Chemical contaminants include metals, chlorinated pesticides, oil, fuel, soot, and butyltins, amongst others (National Oceanic and Atmospheric Administration, 2016). The United States Environmental Protection Agency lists 126 Priority Pollutants in industrial discharges, in addition to the many emerging contaminants of concern (United States Environmental Protection Agency, 2014; Hutchinson et al., 2013). But it is untreated sewage in coastal waters that is of primary concern because it often contains pathogens that can cause illness from a single exposure (Shuval, 2003; Gersberg et al., 2006; Griffin et al., 2003). Pathogenic viruses cause most cases of illness from contact with sewage contaminated waters (Shuval, 2003; Griffin et al., 2003). Serious illness can result from exposure to low numbers of viruses (i.e. 1’s–10’s) which have been shown to remain infectious in seawater longer than bacteria (Gersberg et al., 2006; Griffin et al., 2003; Schiff et al., 1983; Fong & Lipp, 2005; Munn, 2011). Over 100 enteric viruses—those affecting the gut—have been detected in contaminated waters, including hepatitis A virus (HAV) and norovirus, and SARS-CoV-2 virus is present in sewage (Gersberg et al., 2006; Munn, 2011; Lodder & de Roda Husman, 2020). Globally, over 120 million cases of gastrointestinal disease and more than 50 million cases of respiratory disease are estimated to be caused each year by people entering contaminated coastal waters (Shuval, 2003).

Coastal water quality is an increasing challenge for developed and developing countries alike. San Diego County, CA, USA, has 80 km of coastline, much of which is impacted by seasonal stormwater runoff that enters the ocean untreated, a common occurrence in coastal cities worldwide (Gersberg et al., 2006; Griffin et al., 2003; Dwight et al., 2011; Steele et al., 2018). Human fecal pollution associated with stormwater runoff has been documented at San Diego beaches (Gersberg et al., 2006; Steele et al., 2018). The problem is compounded at Imperial Beach, San Diego County’s most southwesterly city, which lies across the border from Tijuana, Mexico. Rainfall in Tijuana River watershed results in both stormwater and sewage being discharged untreated into the Tijuana River, which empties into the ocean 2 km north of the US/Mexico border and pollutes the coastal waters on both sides. Specific infrastructure failures have resulted in additional releases of untreated sewage in recent years (Dibble & Smith, 2017; Hernandez, 2017). Moreover, other point sources of untreated sewage further south broaden the problem. The health risks are significant, with HAV having been detected in Imperial Beach’s coastal waters following rainfall (Gersberg et al., 2006). Beach water quality is monitored in San Diego on a weekly basis; when poor water quality is detected, beaches are closed to direct water contact (i.e. swimming, surfing) (County of San Diego Department of Environmental Health and Quality, 2017). But other forms of exposure to polluted water are possible, including airborne exposure, as we aim to demonstrate in this study.

Sea spray aerosol (SSA) produced by breaking waves in the open ocean and surfzone transfers microscopic droplets of seawater into the atmosphere (Lewis & Schwartz, 2004; Lenain & Melville, 2017; Gantt & Meskhidze, 2013). Sea spray aerosol comprises a significant fraction of total aerosol at the coast and 20+ km downwind, especially during elevated winds, whitecaps, and surf (Clarke, Owens & Zhou, 2006; Van Eijk et al., 2011; de Leeuw et al., 2000). The majority of SSA particles have diameters ranging between 10’s of nm to 10 µm (Fröhlich-Nowoisky et al., 2016). SSA particles contain various chemical compounds and microorganisms, including bacteria and viruses (Quinn et al., 2015; Patterson et al., 2016). Airborne microorganisms have been found to be most abundant in SSA particles greater than 2 µm in diameter (Patterson et al., 2016; Shaffer & Lighthart, 1997; Montero, Dueker & O’Mullan, 2016). Genetic sequencing efforts have shown that coastal air contains a mixture of microorganisms from the ocean and land (Li et al., 2011; Urbano et al., 2011; Graham et al., 2018). Culturing approaches have demonstrated the presence of viable bacteria and viruses in coastal and marine aerosol (Shaffer & Lighthart, 1997; Li et al., 2011; Urbano et al., 2011; Baylor et al., 1977; Fahlgren et al., 2010; Smith et al., 2013; Ladino et al., 2019). Hazardous chemicals and microorganisms have been detected in SSA (Graham et al., 2018; Kirkpatrick et al., 2010; Fleming et al., 2011; Cheng et al., 2005; Pierce et al., 2003; Michaud et al., 2018). The detection of brevetoxins in coastal air downwind of harmful algal blooms confirmed reports of naturally occurring marine toxins reaching humans on land via airborne transport (Kirkpatrick et al., 2010; Fleming et al., 2011; Cheng et al., 2005; Pierce et al., 2003). Genetic sequencing of coastal aerosol has identified potentially pathogenic bacteria at the species and genera levels (Graham et al., 2018). Whole genome shotgun sequencing identified potentially pathogenic bacteria in isolated SSA (Michaud et al., 2018).

Coastal water pollution has the potential to transfer into the atmosphere in SSA and reach people inland, a growing public health concern (O’Mullan, Dueker & Juhl, 2017). Baylor and colleagues demonstrated sea-to-air virus transfer by releasing non-native viruses into coastal waters and recovering the same virus strains on the beach (Baylor et al., 1977). Exposure to aerosolized microorganisms has also been identified as a risk in other environments, including solid waste treatment facilities, wastewater treatment plants, and agricultural sites using wastewater for spray irrigation (Fannin et al., 1977; Teltsch & Katzenelson, 1978; Carducci, Arrighi & Ruschi, 1995; Li et al., 2021; Malakootian et al., 2013; Brisebois et al., 2018). This risk has been demonstrated for aerosols from sewage impacted rivers using aeration remediation (Dueker et al., 2012; Dueker & O’Mullan, 2014). Aerosols released into the air from rivers have been linked with illness (Pickup et al., 2005). The objectives of the present study were to: (a) use a nontoxic dye to simulate large-scale coastal water pollution events; (b) determine the conditions leading to the transfer of the dye to the atmosphere; and (c) relate dye concentrations between the ocean and atmosphere. This study is the first to examine the potential for inland transport of airborne water pollution in coastal regions. The detection of the dye in the air inland from the beach demonstrates that coastal water pollution transfers to the air in SSA and reaches communities via an airborne exposure pathway.

## Materials & methods

### Tracer dye as a water pollution mimic

**Figure 1:**
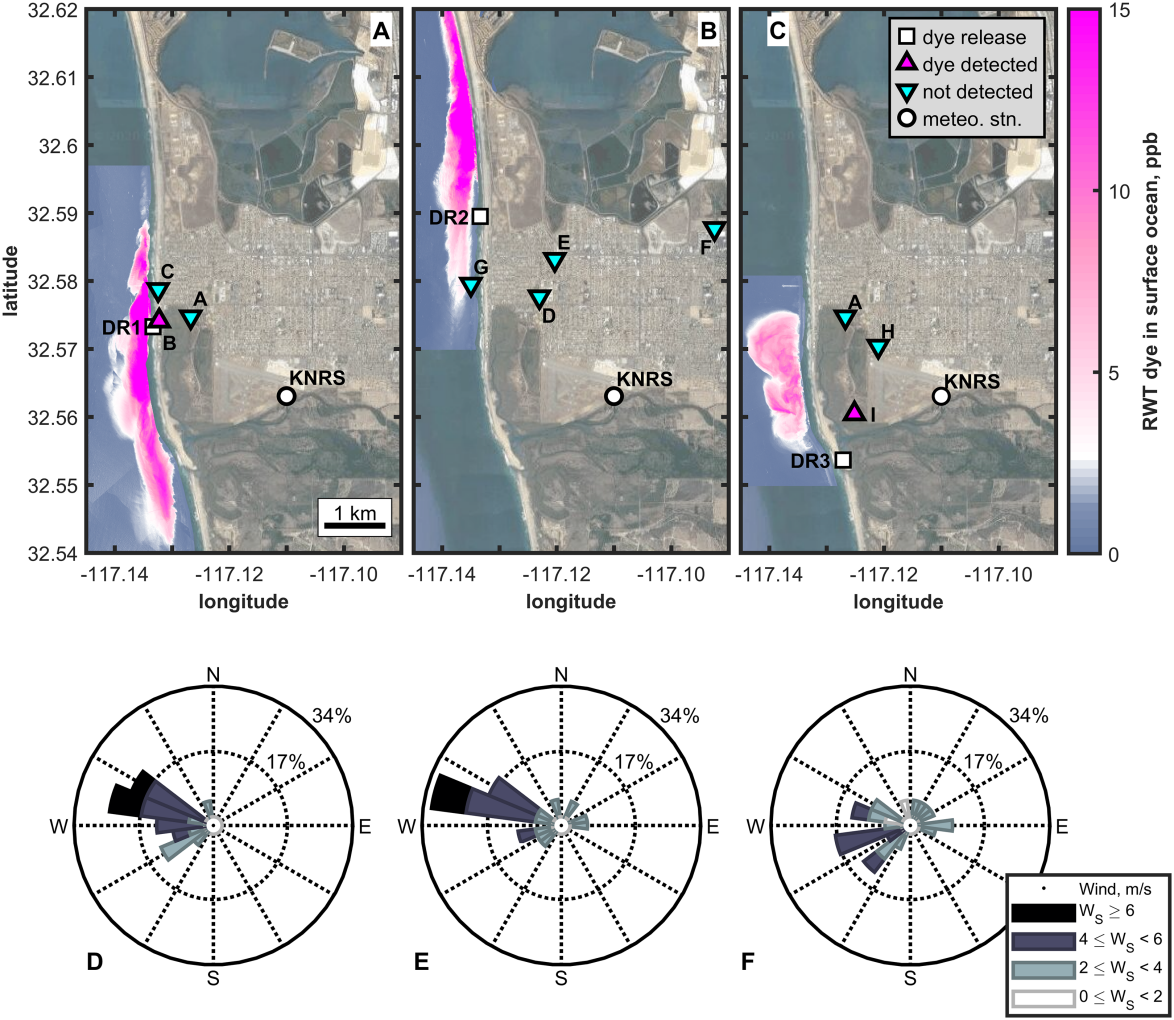
Overview of the three dye releases of the CSIDE study. (A)–(C) present maximum RWT dye concentrations in coastal waters and aerosol sampling locations and results from each dye release (1–3). Magenta triangles pointing up indicate locations where dye was detected in the aerosol. Cyan triangles pointing down show the locations where aerosol was sampled and dye was not detected. (D)–(F) contain wind roses presenting daytime winds, responsible for onshore transport, for the first 2 days of each DR, when most aerosol samples were collected (4). Stacked bars in the wind roses indicate the percent of the time period the wind blew from the indicated direction and at the indicated speed (represented by color). (1) Zohar Bar-Yehuda (2017). zoharby/plot_google_map (https://github.com/zoharby/plot_google_map), GitHub. Retrieved June 1, 2017. (2) Map data: Imagery ©2020 Google. Data USGS, Data SIO, NOAA, U.S. Navy, NGA, GEBCO, Data LDEO-Columbia, NSF, NOAA, Imagery ©2020 TerraMetrics, Map data ©2020 INEGI. (3) Jonathan Sullivan (2017). Automatic Map ScaleGeneration (https://www.mathworks.com/matlabcentral/fileexchange/33545-automatic-map-scale-generation), MATLAB Central File Exchange. Retrieved June 1, 2017. (4) Daniel Pereira (2017). Wind Rose (https://www.mathworks.com/matlabcentral/fileexchange/47248-wind-rose), MATLAB Central File Exchange. Retrieved June 1, 2017.

The CSIDE study simulated the impacts of coastal water pollution events on coastal air quality by releasing rhodamine WT (RWT) dye into the surfzone at Imperial Beach, CA, USA (Fig. 1) (Grimes et al., 2020). Rhodamine WT (molecular weight 566.99 g/mol) is a water soluble, non-toxic, fluorescent dye used as a semi-conservative water mass tracer, having no known oceanic sources and a minor sink in the form of photodegradation (Smart & Laidlaw, 1977; Wilson, Cobb & Kilpatrick, 1986; Suijlen & Buyse, 1994; Clark et al., 2009). It is a non-volatile, solid compound at room temperature with maximum fluorescence at excitation/emission wavelengths of 558/582 nm (Wilson, Cobb & Kilpatrick, 1986; Mackay & Shiu, 1981). We simulated three pollution events by releasing roughly 30 gallons of concentrated RWT solution into the surfzone on September 23 (early morning, 3.2 h release), October 8 (early morning, 3.8 h release), and October 12 (mid morning, 1.8 h release into estuary outflow on ebb tide), 2015 (Fig. 1). Sea surface concentrations of the fluorescent dye (Fig. 1, Table 1) were measured with a hyperspectral camera mounted on an airplane that flew over the study site multiple times each hour on the first 2 days of each dye release (DR) (Melville et al., 2016). The hyperspectral camera was calibrated with in-situ near-surface dye measurements to provide dye concentrations in surface waters with high spatial resolution (2 m × 2 m) (Grimes et al., 2020; Melville et al., 2016; Clark et al., 2014; Feddersen et al., 2016). This method is limited to daylight hours, and therefore no nighttime sea surface dye concentrations are reported.

**Table 1:** Data for the aerosol samples collected following the three dye releases. Distance inland is the distance from the air sampling location to the nearest point along the shoreline. Distance downwind is the mean distance along the wind vector from the center of the 200 m × 100 m ocean source window to the aerosol sampling site. Distance inland is fixed, whereas distance downwind is subject to the relevant wind directions. [dye]_air_ (pg/m^3^) is the concentration of dye in the air calculated from the fluorescence intensity and the volume of air sampled. The four samples which match our criteria for dye detection are highlighted in bold. The values for the samples that did not meet our criteria for dye detection are provided in parentheses. [dye]_sea_ (ppb) indicates the sea surface dye concentration measured by the hyperspectral camera and are available for most daylight hours on the day of each dye release and the following day and are not available for aerosol samples collected at night.

#	DR	date start	sample period	Site	inland (m)	downwind (m)	sampled air (m^3^)	dye pattern	above LoD	[dye]_air_ (pg/m^3^)	[dye]_sea_, (ppb)
1	1	9/23	day	A	615	678	198	Yes	No	(14.3)	4.4
**2**	**1**	**9/23**	**day**	**B**	**101**	**154**	**135**	**Yes**	**Yes**	**759.0**	**3.6**
3	1	9/23	night	A	615	653	398	No	No	(4.8)	0.6
4	1	9/24	day	A	615	664	226	No	No	(5.2)	0.7
**5**	**1**	**9/24**	**day**	**B**	**101**	**148**	**162**	**Yes**	**Yes**	**43.3**	**0.8**
6	1	9/24	night	C	122	NA	422	No	No	(5.0)	NA
7	1	9/24	night	A	615	NA	377	No	No	(11.3)	NA
8	1	9/25	day	C	122	NA	116	No	No	(20.0)	NA
9	1	9/25	day	A	615	NA	137	No	No	(4.8)	NA
10	2	10/8	day	D	976	1057	241	No	No	(8.3)	0.5
11	2	10/8	day	E	1256	1310	216	No	No	(9.4)	0.6
12	2	10/8	day	F	3867	3953	181	No	No	(8.9)	3.4
13	2	10/8	night	E	1256	1284	428	No	No	(12.5)	0.3
14	2	10/8	day	D	976	1037	54	No	No	(20.4)	0.3
15	2	10/9	day	G	108	57	140	No	No	(9.2)	0.4
16	2	10/9	24 h	E	1256	1288	691	No	No	(5.3)	0.5
17	3	10/12	day	A	615	712	211	No	Yes	(44.6)	1.8
18	3	10/12	day	H	1193	1275	234	No	No	(14.1)	2.6
**19**	**3**	**10/12**	**day**	**I**	**668**	**720**	**178**	**Yes**	**Yes**	**184.2**	**0.6**
20	3	10/12	night	H	1193	NA	347	No	No	(15.2)	NA
21	3	10/12	night	A	615	NA	376	No	No	(16.4)	1.0
**22**	**3**	**10/13**	**day**	**I**	**668**	**694**	**169**	**Yes**	**Yes**	**55.5**	**0.1**
23	3	10/14	day	I	668	NA	135	No	Yes	(60.9)	NA
24	3	10/15	day	I	668	NA	152	No	Yes	(34.0)	NA
25	blank	10/7	night	D	976	NA	192	No	NA	(2.0)	NA
26	blank	10/7	night	C	122	NA	383	No	NA	(7.3)	NA
27	blank	10/7	day	D	976	NA	119	No	NA	(18.9)	NA
28	blank	10/7	day	C	122	NA	224	No	NA	(10.0)	NA
29	rinse	9/23	NA	rinse	NA	NA	NA	No	NA	NA	NA
30	rinse	10/9	NA	rinse	NA	NA	NA	No	NA	NA	NA
								**blanks mean**		**9.5**	
								**blanks SD**		**7.1**	
								**LoD**		**32.8**	

### Aerosol sampling

Atmospheric aerosols were sampled at up to 3 sites over 2–4 days following each DR, at a total of 9 locations (Fig. 1 and Table 1). Aerosol sampling began the morning of each DR and continued into the afternoon, when samples were recovered and new sampling periods began, yielding ~6 h daytime and ~16 h overnight collection periods. Aerosol sampling locations were selected each morning based on the current dye observations (aerial and in-situ) in an effort to sample downwind of the ocean dye. The oceanic dye plume continued to evolve over time such that the aerosol sampling sites did not always remain downwind, and the downwind distance varied. Aerosols were collected using a PAS450-10 SpinCon I (heretofore “SpinCon I”; see Supplemental Materials for more information), which utilizes a swirling technique to transfer aerosols and water soluble compounds from the air into a fixed volume of sterile water (Michaud et al., 2018; Yooseph et al., 2013). The SpinCon I samples air at 450 lpm and can sample continuously for hours, greatly concentrating airborne constituents into a 10 ml liquid sample. Internal surfaces of the SpinCon I were cleaned with 70% ethanol and tubing was flushed with 10% bleach followed by sodium thiosulfate to neutralize the bleach. In between samples, rinse cycles, in which the instrument is run for 30 s and then the sample is discarded, were used to flush surfaces. Field blanks were collected on October 7–8, between the first and second DRs, after the dye had dissipated in the coastal waters for 2 weeks.

### RWT measurement in aerosol samples

Samples were analyzed for RWT using fluorescence spectroscopy, normalizing to the water Raman peak (Lawaetz & Stedmon, 2009; Murphy, 2011). The fluorescence measurements were made the day the samples were recovered, using the Horiba Aqualog fluorescence spectrometer (Aqualog UV 800C, Horiba Ltd., Kyoto, Japan), at 425–625 nm excitation wavelengths, at 5 nm increments, and reading at emission wavelengths of 248–827 nm, at ~4.5 nm increments. A calibration curve was generated to quantitatively relate fluorescence to RWT concentration, which also provided the RWT fluorescence pattern in the excitation-emission matrix, which we used to identify RWT in aerosol samples (Fig. S1). Positive detection of RWT in the aerosol samples are those that showed the RWT fluorescence pattern and a RWT fluorescence intensity above that of our limit of detection derived from the field blanks (Eq. 1) (Armbruster & Pry, 2008):

(1) limitofdetection=α−3.29β

where α is the mean of the field blanks and β is the standard deviation of the field blanks. The RWT fluorescence pattern was not detected in any aerosol field blanks nor any sampler rinses.

### Atmospheric dye concentrations

The average concentration of RWT in the air during each sampling period was determined by dividing the amount of RWT in the SpinCon I liquid sample by the total volume of air sampled. Figure S2 shows fluorescence spectra from a RWT standard (Fig. S2A), from aerosol samples with strong and weak RWT signatures (Figs. S2B & S2C), and from a field blank (Fig. S2D), as well as the peaks used for quantitation and the region for background correction. See Supplemental Information. RWT concentrations are reported for all air samples and those which met both criteria for RWT detection are noted (Table 1).

### Dye concentrations in upwind waters

To determine sea surface dye concentrations upwind of the aerosol samplers ([dye]_sea_ (ppb); Table 1; *x*-axis in Fig. 3), wind vectors were used from a local meteorological station (KNRS, Fig. 1) and interpolated to the time intervals of aerial sea surface dye measurements. No lag between source and wind times was imposed because the observed ~5 m/s winds advected material from the surfzone to the furthest sampler (~1.5 km) in ~5 min, much shorter than the multi-hour sampling intervals. The intersection of the interpolated upwind vector with the tidally varying shoreline location, estimated using the 2012 NOAA Tsunami DEM and local water-level (including tides, but neglecting wave set-up), provided the center location for the upwind dye source in the ocean. A 200 m alongshore by 100 m offshore sea surface dye concentration window was extracted for each sea surface dye measurement taken during each aerosol sampling period. This box was limited to 100 m in the offshore direction because SSA was mainly generated in the surfzone region of depth-limited breaking waves (Van Eijk et al., 2011; de Leeuw et al., 2000). The spatial and temporal average of the sea surface dye concentration in those windows was taken for each aerosol sampling period ([dye]_sea_ (ppb)). This allowed us to precisely define the RWT aerosol source waters and quantitatively relate RWT concentrations in the ocean and atmosphere.

## Results

### RWT transport by ocean currents

The three DRs demonstrated different ocean transport pathways of the RWT, driven by the coastal ocean currents. Figures 1A–1C show the maximum sea surface dye concentration at each point in the ocean on the first day of each dye release as measured from the air with the hyperspectral camera (Melville et al., 2016; Clark et al., 2014). Although temporal information such as persistence/transience is obscured in these figures, they provide a broad visual comparison of the major dye distribution for each DR. At the broadest level, the three DRs showed that the pollution mimic did not immediately disperse offshore, but instead remained within 1 km of the shoreline during the first day of each release, consistent with multiple studies showing surfzone trapping (Boehm, 2003; Grant et al., 2005; Clark, Feddersen & Guza, 2010; Rodriguez, Giddings & Kumar, 2018). For DRs 1 and 3 (Figs. 1A & 1C), the dye remained within ~2 km alongshore of the release location. For DR2 (Fig. 1B) a strong, wave-driven, alongshore current rapidly advected the dye ~10 km northward along the coast (Grimes et al., 2020; Wu et al., 2020). Thus for DR2, although high sea surface dye concentrations are seen along the coast, they only persisted at any given location for a short time due to the rapid northward advection. Sea surface dye concentrations are overall much lower for DR3, likely a result of localized mixing and, to a lesser extent, missing aerial measurements due to flight access limitations in the first few hours following the DR. Combined, the three DRs show the variability in water and pollution transport expected for the coastal ocean and the motivation for the CSIDE study. The ocean transport aspects of the CSIDE study are discussed in greater depth in other publications (Grimes et al., 2020; Wu et al., 2020).

### RWT detected in coastal aerosols

Dye was detected in coastal aerosols on days 1 and 2 of DRs 1 and 3 in four total aerosol samples (Figs. 1A & 1C, Table 1). Dye was detected as far as 668 m inland and 720 m downwind from source waters. Wind rose plots (Figs. 1D–1F) for daytime winds show that onshore winds, typical of coastal meteorology patterns, were common. Wind data link the oceanic source waters with locations of atmospheric dye detection over land. Figure 1 broadly shows that atmospheric dye was detected downwind of the dye plume. The well characterized distribution of RWT in coastal waters allows quantitative comparison between dye concentrations in the air and in the source waters.

### Comparing ocean and atmospheric observations

**Figure 2:**
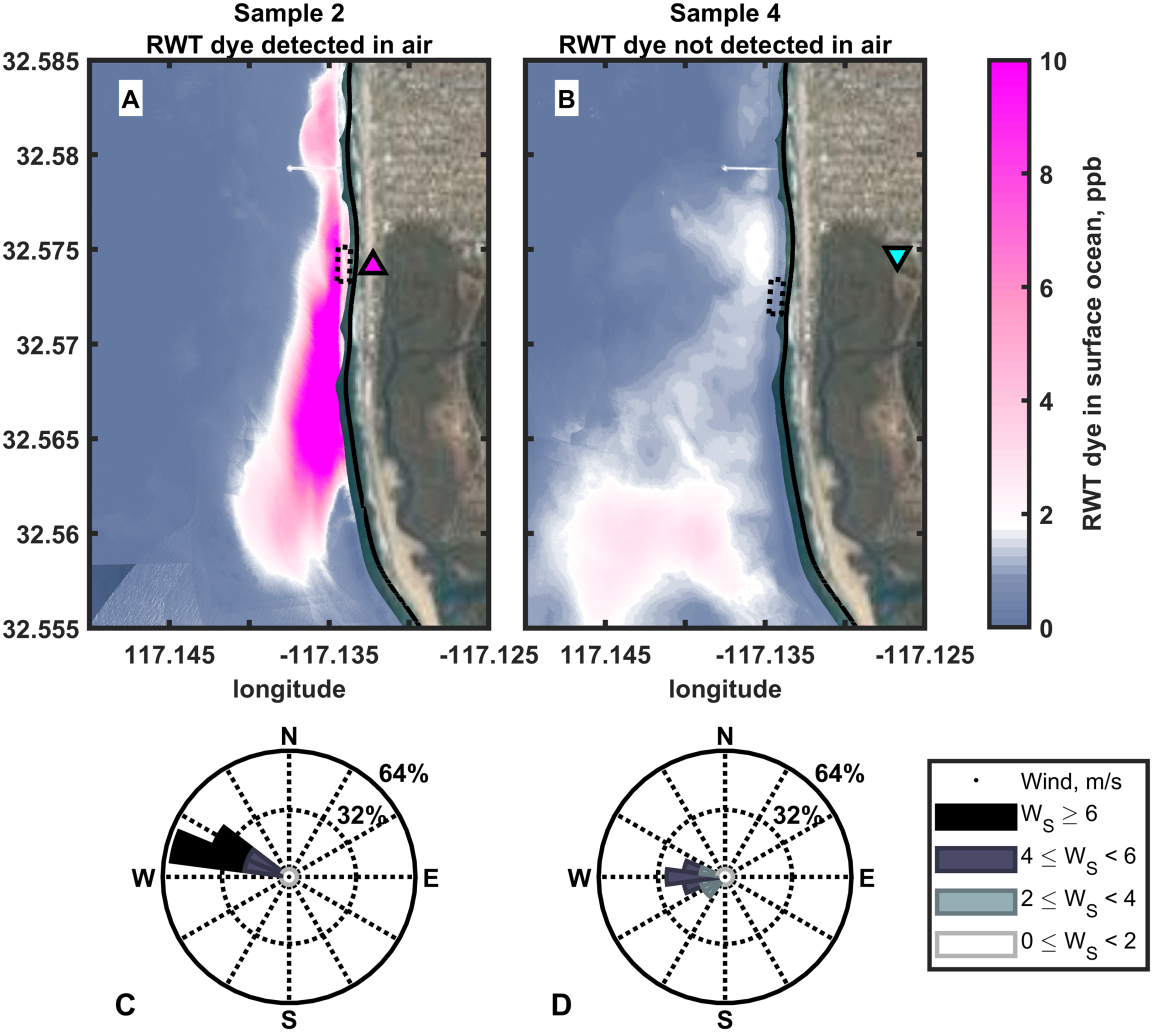
Mean sea surface dye concentrations upwind of aerosol sampling locations. (A) and (B) show average sea surface dye concentrations during the period each aerosol sample was collected at the location on land indicated by the triangle (5–7). The 200 m × 100 m dotted black box indicates where the average wind direction during each sampling interval intersected the coastal waters from the sampling site. Figure 3 finds the location of that box and its mean dye value for each hourly sea surface dye measurement. Here we show the mean dye field during each air sampling period and the mean upwind location (box). (C) and (D) are wind roses for the winds observed at the KNRS meteorological station during the aerosol sampling periods (8). Aerosol sample 2 (A & C) contained dye and was collected downwind of high dye concentrations in coastal water. Aerosol sample 4 (B & D) did not contain detectable dye and was collected downwind of seawater containing very low dye concentrations near the detection limit. (5) Zohar Bar-Yehuda (2017). zoharby/plot_google_map (https://github.com/zoharby/plot_google_map), GitHub. Retrieved June 1, 2017. (6) Map data: Imagery ©2020 Google. Data USGS, Data SIO, NOAA, U.S. Navy, NGA, GEBCO, Data LDEO-Columbia, NSF, NOAA, Imagery ©2020 TerraMetrics, Map data ©2020 INEGI. (7) Jonathan Sullivan (2017). Automatic Map ScaleGeneration (https://www.mathworks.com/matlabcentral/fileexchange/33545-automatic-map-scale-generation), MATLAB Central File Exchange. Retrieved June 1, 2017. (8) Daniel Pereira (2017). Wind Rose (https://www.mathworks.com/matlabcentral/fileexchange/47248-wind-rose), MATLAB Central File Exchange. Retrieved June 1, 2017.

Repeated measurements of dye in the coastal waters allow us to examine dye concentrations in the source waters during the atmospheric sampling periods. Figure 2 shows the comparison of ocean dye data from two exemplary samples for when dye was and was not detected in the air. The mean sea surface dye concentration in the upwind waters during the aerosol sampling period is presented relative to the aerosol sampling location on land. The inset dotted box shows a 200 m × 100 m region centered where the mean wind direction during the aerosol sampling period intersected the coastal waters. Sample 2 (left panel) was collected on the first day of DR1 and sample 4 (right panel) was collected the day after, i.e. day 2 of DR1. Dye was detected in sample 2, which was collected downwind of waters with high dye concentrations. Dye was not detected in sample 4, which was collected downwind of waters with little to no detectable dye. Analogous plots for all air samples with coupled ocean measurements are provided in the Supplemental Materials (Fig. S3).

**Figure 3:**
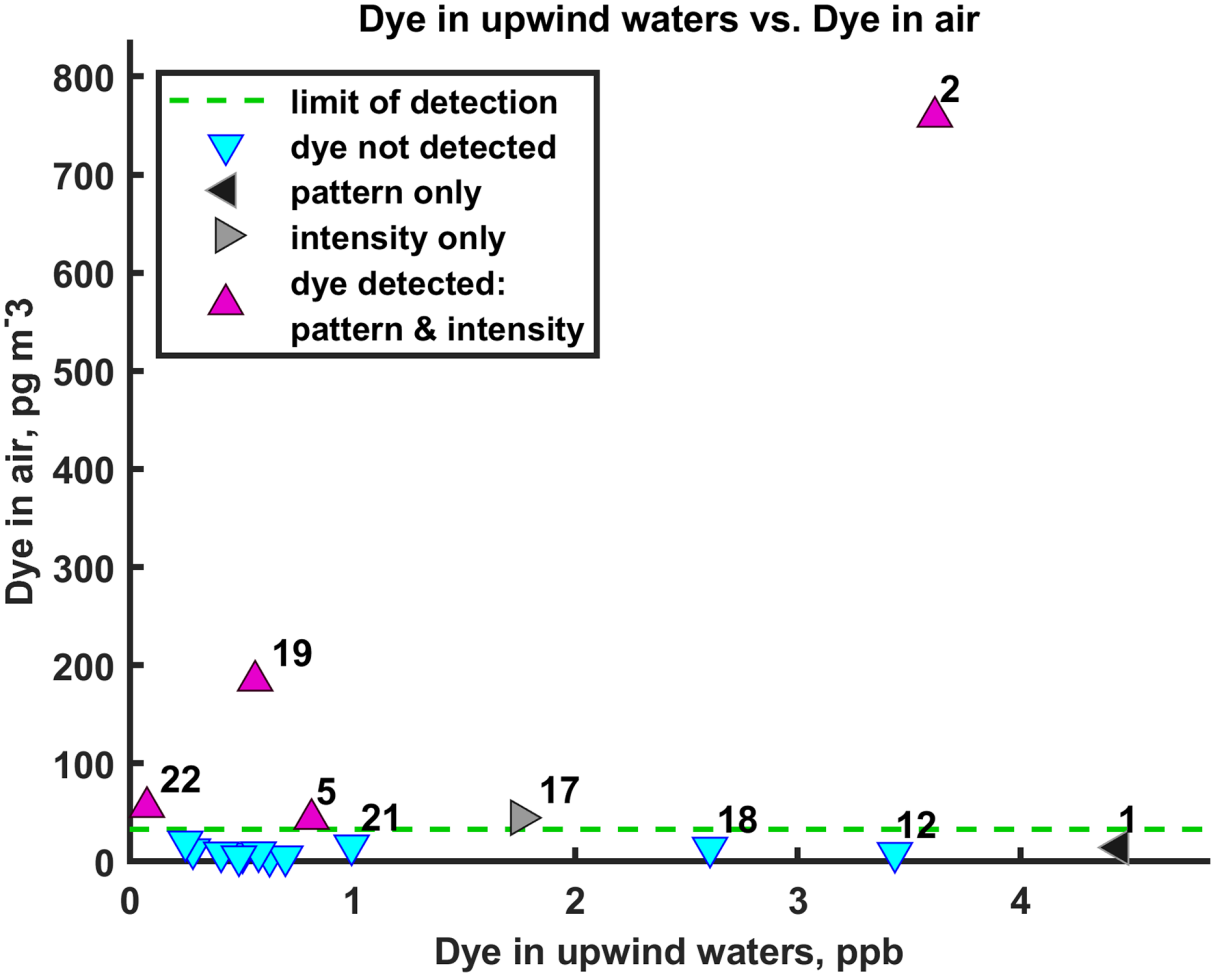
Dye concentrations in air and upwind waters. For aerosol samples that were collected during periods of hyperspectral measurements of ocean dye concentrations (primarily daytime), the RWT concentration in the air (vertical axis) is compared against the dye concentration in the upwind source waters (horizontal axis). Symbol labels are sample numbers. A dye concentration in the air can be calculated whether dye was detected (magenta) or not (cyan) as dye detection relies both on values being above the blank and containing the RWT signature. The green dashed line indicates the limit of detection determined from the air field blanks and Eq. (1) (32.8 pg/m^3^). False positives would show up along a vertical line near 0 on the horizontal axis. Here, two air samples met one of two criteria for dye detection and two other air samples did as well, but do not appear here because sea surface RWT concentrations are not available for them.

## Discussion

### Atmospheric transfer of RWT in SSA

Surfzone production of SSA transferred RWT from the ocean to the atmosphere. Rhodamine WT’s transfer in SSA is consistent with the detection of other chemical species in SSA (Fleming et al., 2011; Cheng et al., 2005; Hawkins & Russell, 2010; Cochran et al., 2017; Kuznetsova, Lee & Aller, 2005) and larger entities including viruses and bacteria (Patterson et al., 2016; Baylor et al., 1977; Michaud et al., 2018; Aller et al., 2005). Dissolved RWT has a low surface activity, therefore it acts as a tracer of bulk seawater, which transfers to SSA primarily in jet drops. Sea spray aerosol production, specifically from depth-limited waves breaking in the surfzone, must have been the dominant RWT transfer mechanism because surfzone production of SSA has been shown to be dominant for low to moderate wind speeds (Van Eijk et al., 2011; de Leeuw et al., 2000). Dye transfer outside the surfzone was negligible because whitecap conditions were not common during the study period, due to low wind speeds often below 6 m/s.

### Atmospheric transport of RWT SSA

We understand that the major factors influencing atmospheric concentrations of RWT SSA include: dye concentrations in aerosol source waters, which depends on advection and dispersion of the dye in the ocean, driven by waves, regional pressure gradients, buoyant plumes, winds, tides, and internal tides; ocean-to-atmosphere RWT SSA flux, which occurred primarily through SSA production by surfzone depth-limited wave breaking; downwind advection and horizontal and vertical dispersion in the atmosphere, driven by coastal winds and atmospheric boundary layer dynamics; and dry deposition (gravitational settling) of RWT SSA. We understand that dye was not detected in most aerosol samples due to high variability in ocean and air transport patterns. Photobleaching of RWT is negligible on our <4 d timescales (Suijlen & Buyse, 1994). We compare dye concentrations in the ocean and atmosphere to test whether source water dye concentrations influenced airborne dye concentrations (Fig. 3). Dye was not always detected when upwind waters contained elevated dye concentrations, due to downwind distance, 3D atmospheric dispersion, and the lack of a vertical sampling array. When RWT dye was detected in the aerosol (Fig. 3, magenta points), the data suggest dye concentrations in the air are proportional to dye concentrations in source waters. Importantly, there were no false positive atmospheric dye values because higher RWT concentrations in the air correspond to higher RWT concentrations in the ocean; there are no data points high in air RWT and low in ocean RWT (Fig. 3). More experiments with sampling at higher temporal and vertical/horizontal spatial resolution are needed to confirm a robust, quantitative relationship between SSA and upwind source waters, and to better understand the role of atmospheric dispersion.

### Implications

Our results demonstrate the ability to trace SSA back to its source waters with high spatial and temporal resolution, and to quantitatively relate dye concentrations in source waters and air masses. More importantly, this study confirms coastal water pollution can be transferred from the surf into the atmosphere by releasing a tracer dye into the ocean and detecting it in the air inland from the beach. These observations highlight an under-appreciated airborne exposure pathway to coastal water pollution. We expand on the pioneering work of Baylor et al. (1977) by simulating large-scale artificial water pollution events, measuring sea surface dye concentrations at high spatio-temporal resolution (2 m × 2 m, hourly), tightly constraining RWT SSA source waters, and making quantitative ocean-atmosphere comparisons that extend inland beyond the beach. Whereas many atmospheric aerosol studies detect particles from distant sources, and aerosol dispersion models provide insight into particle origin at regional level resolution (~100 km), we were able to pinpoint SSA source waters on the <1 km scale. Although we were unable to detect RWT SSA beyond 668 m inland (Fig. 1, Table 1), it is well documented that SSA can travel much further (Smith et al., 2013; Bondy et al., 2017; Prospero et al., 2005; Smith et al., 2012). A 2 µm SSA particle has a predicted dry deposition residence time of ~1.5 weeks with an estimated travel distance of 10,000 km (Lewis & Schwartz, 2004). SSA particles this size have been detected hundreds to thousands of km from their oceanic source region (Bondy et al., 2017). Bacteria can remain viable after travelling thousands of km in the atmosphere (Prospero et al., 2005; Smith et al., 2012). Our findings emphasize that species transfer from the ocean to the atmosphere includes not just natural seawater components but also pollutants. This study also demonstrates that coastal water pollution reaches people beyond the shoreline through atmospheric transport. These results detail a promising approach for linking pollution in source waters, including the surfzone, estuaries, and other outflows, to atmospheric pollutants and health impacts downwind.

## Conclusions

In summary, this study confirms that coastal water pollution can be transferred from the surf to the atmosphere by measuring tracer dye concentrations in the ocean and air. Airborne transport of coastal water pollution can expose people beyond the water and beach, including entire coastal communities, to biological and chemical pollutants. The magnitude of this risk is a function of various parameters. Dispersion in the atmosphere dilutes airborne pollution, and pathogens can die or deactivate in the atmosphere, but exposure to a relatively small number of viruses have been shown to cause illness (Schiff et al., 1983; Fong & Lipp, 2005; Munn, 2011). This study draws attention to an underappreciated airborne exposure pathway and sets the stage for future studies to better quantify the magnitude of the airborne exposure. Additional work is needed to assess public health threats from known coastal pathogens and emerging pathogens, like SARS-CoV-2, which is present in wastewater (Lodder & de Roda Husman, 2020; Colwell, 1996). Coastal water pollution is an increasing global problem that will only worsen as the human population grows and climate change leads to more extreme precipitation events. Ultimately, a thorough understanding of how pathogens and toxins are transported by coastal water currents and wind patterns will allow timely predictions of potential health risks to coastal communities and beyond.

## Supplemental Information

10.7717/peerj.11358/supp-1 Supplemental Information 1 RWT fluorescence calibration curve for aerosol measurements. Calibration curve produced by measuring the fluorescence of RWT solutions at known concentrations. The data were background corrected using the same technique used for the collected samples.

10.7717/peerj.11358/supp-2 Supplemental Information 2 Example excitation-emission matrices (EEMs) displaying fluorescence intensity in Raman Units (RU). (A) a 2 PPB RWT standard, (B) sample #19 - dye detected, (C) sample #20 - dye not detected, and (D) sample #27 - aerosol field blank; dye not detected Red dots indicate the 3 excitation/emission pairs determined from the calibration to be used for RWT dye quantification. The mean fluorescence intensity from the black rectangle was subtracted from the entire spectrum as an internal background correction.

10.7717/peerj.11358/supp-3 Supplemental Information 3 Air sampler manual from manufacturer.

10.7717/peerj.11358/supp-4 Supplemental Information 4 Mean sea surface dye concentrations upwind of aerosol sampling locations. Like Figure 2, for all aerosol samples with data available (day samples). Right: Average sea surface dye concentrations during the period each aerosol sample was collected at the location on land indicated by the triangle. The 200 m × 100 m dotted box indicates where the average wind direction during each sampling interval intersected the coastal waters from the sampling site. Figure 3 finds the location of that box and its mean dye value for each hourly sea surface dye measurement. Here we show the mean dye field during each air sampling period and the mean upwind location (box). Left: wind rose for the winds observed at the KNRS meteorological station during the aerosol sampling periods.

10.7717/peerj.11358/supp-5 Supplemental Information 5 Supplemental Methods. More details on methods used

## Additional Information and Declarations

### Data Availability

The following information was supplied regarding data availability: Pendergraft, Matthew A.; Grimes, Derek J.; Giddings, Sarah N.; Feddersen, Falk; Beall, Charlotte M.; Lee, Christopher; Santander, Mitchell V.; Prather, Kimberly A. (2021). Data from: Airborne transmission pathway for coastal water pollution. In Center for Aerosol Impacts on Chemistry of the Environment (CAICE). UC San Diego Library Digital Collections. https://doi.org/10.6075/J0V40SSD
