# Supplementary figures and images for: Airborne transmission pathway for coastal water pollution
Source: PeerJ. 2021 Jun 7;9:e11358. doi: 10.7717/peerj.11358 (PMC8191489; doi:10.7717/peerj.11358)

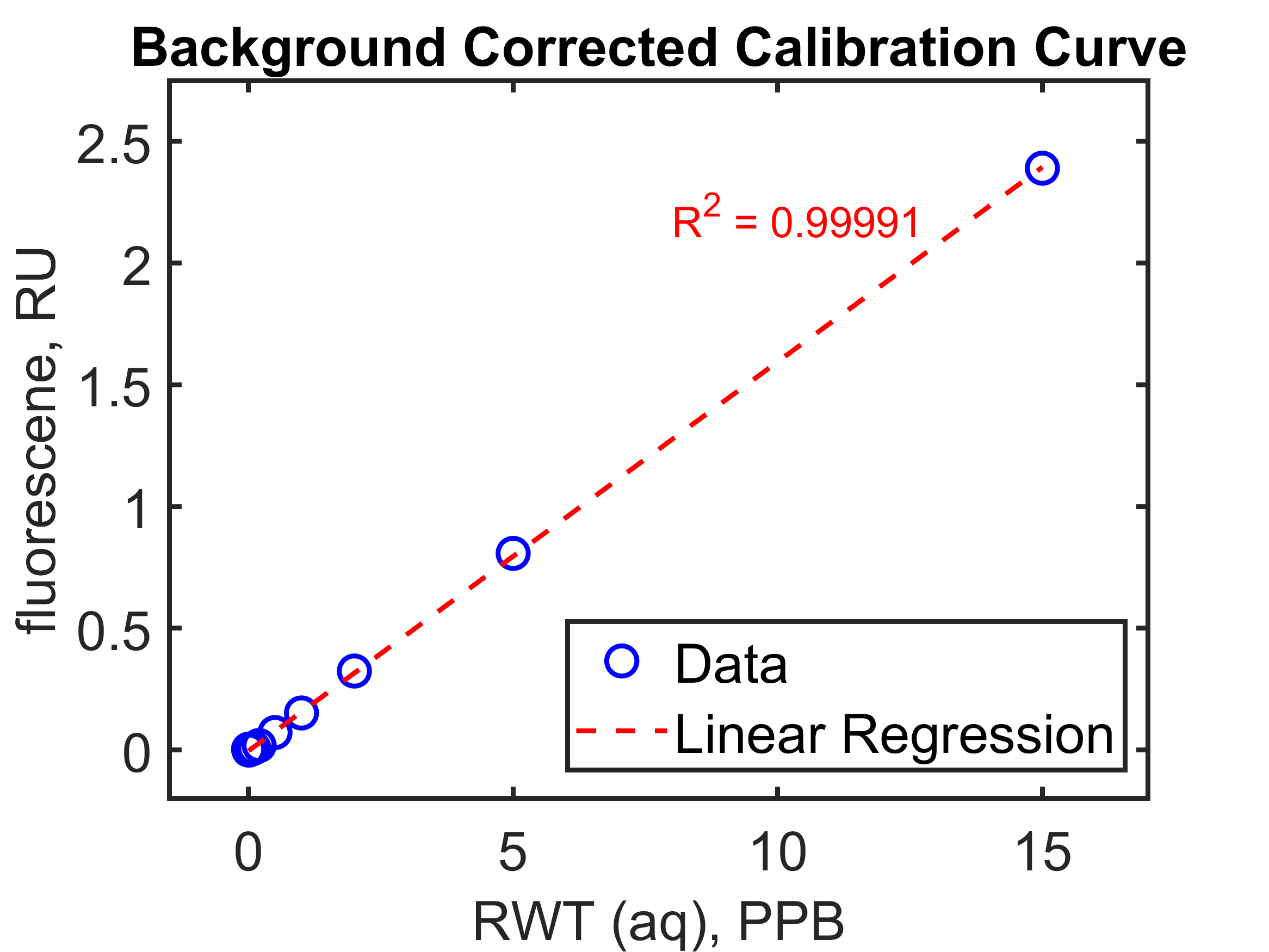

Supplement: Supplemental Information 1 — Calibration curve produced by measuring the fluorescence of RWT solutions at known concentrations. The data were background corrected using the same technique used for the collected samples. [file peerj-09-11358-s001.png]

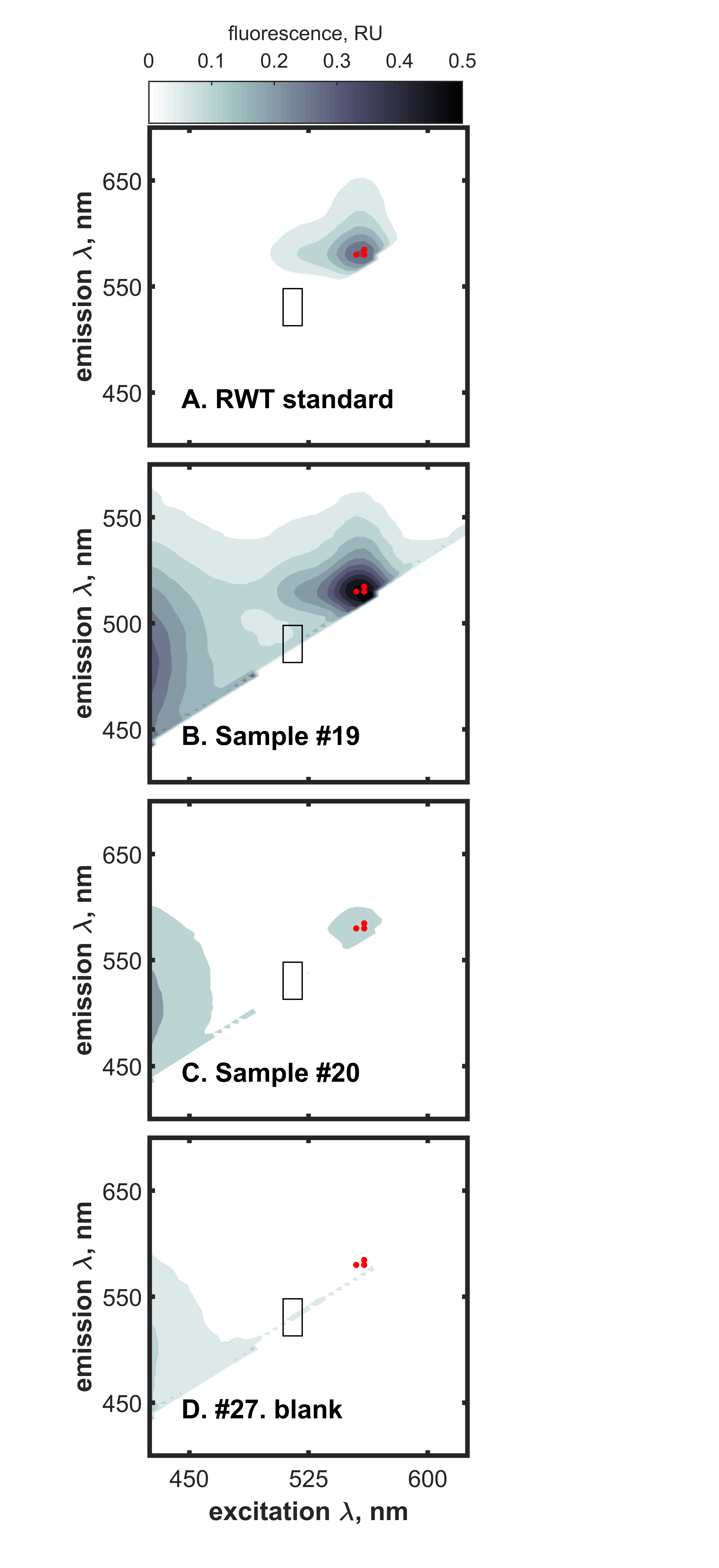

Supplement: Supplemental Information 2 — (A) a 2 PPB RWT standard, (B) sample #19 - dye detected, (C) sample #20 - dye not detected, and (D) sample #27 - aerosol field blank; dye not detected Red dots indicate the 3 excitation/emission pairs determined from the calibration to be used for RWT dye quantification. The mean fluorescence intensity from the black rectangle was subtracted from the entire spectrum as an internal background correction. [file peerj-09-11358-s002.png]
