# Supplementary material for: Airborne transmission pathway for coastal water pollution
Source: PeerJ. 2021 Jun 7;9:e11358. doi: 10.7717/peerj.11358 (PMC8191489; doi:10.7717/peerj.11358)
Supplement: Supplemental Information 3 [file peerj-09-11358-s003.pdf]

INNOVAPREP

SpinCon® PAS 450-10A
Portable Air Sampler

User Manual

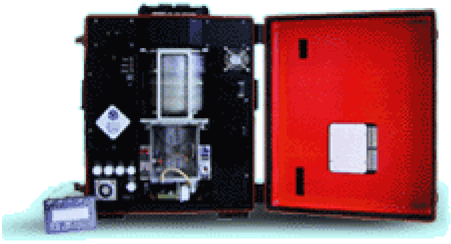

DISCLAIMER OF WARRANTIES

YOU EXPRESSLY UNDERSTAND AND AGREE THAT:

A. INNOVAPREP WARRANTS THAT THE PRODUCT HAS BEEN MANUFACTURED, AND SHALL CONFORM, TO THE PROVIDED SPECIFICATIONS. NO OTHER WARRANTY OR GUARANTEES ARE PROVIDED.

B. YOUR USE OF THE PRODUCT IS AT YOUR SOLE RISK. THE PRODUCT IS PROVIDED ON AN "AS IS" BASIS. INNOVAPREP EXPRESSLY DISCLAIMS ALL WARRANTIES OF ANY KIND, WHETHER EXPRESS OR IMPLIED, INCLUDING, BUT NOT LIMITED TO THE IMPLIED WARRANTIES OF MERCHANTABILITY, FITNESS FOR A PARTICULAR PURPOSE AND NON-INFRINGEMENT.

C. NO ADVICE OR INFORMATION, WHETHER ORAL OR WRITTEN, OBTAINED BY YOU FROM INNOVAPREP, SHALL CREATE ANY WARRANTY NOT EXPRESSLY STATED HEREIN.

TABLE OF CONTENTS

Section 1: Introduction 3
Operating Principle 3
Intended Applications 3
Description 4
Specifications 8
Section 2: Setup and Use 9
Setup 9
Use 10
Shutdown 13
Section 3: Care 15
Operator Maintenance 15
Cleaning External Components 15
Cleaning Internal Fluid Lines 15
Contactor Cleaning 16
Supplies 18
Yearly Preventive Maintenance 18
Section 5: Decontamination 19
Section 5: Environmental Limitations 20
Section 6: Safety Precautions 21
Section 7: Troubleshooting 22
Section 8: Contact Information 23

Section 1: Introduction

The SpinCon® PAS450-10A is a commercially available, portable and energy-efficient wet concentrator air sampler. Tested and proven in user-sponsored tests, it is ideally suited for the collection of bio-aerosols, particulate matter, and soluble vapors, including submicron-sized particles, airborne molecular contamination, biological particulates, and volatile and semi volatile chemical compounds. The system has been developed to address a broad range of advanced air sampling requirements and is rapidly becoming the gold standard for collecting large quantities of airborne pathogens.

Operating Principle

A blower on the SpinCon® PAS 450-10A sampler pulls air through a glass collector/concentrator (C/C) which contains a sampling liquid. As air passes into the C/C, entrained solids impact on the sampling liquid droplets and are captured. Gaseous materials are likewise dissolved in the fluid droplets. The sampling liquid remains in the C/C to sample more and more air. As a result, particles and compounds residing in the air are continuously concentrated in the sampling fluid medium. After the chosen sampling interval, the liquid in the C/C can be drawn off as a discrete liquid sample. This sample can then be analyzed for the constituents of interest using a variety of analytic methods. Compatible analysis methods include:

HAPSITE, FTIR, PCR, Immunoassay Strips, Culturing, Microscopy, Mass Spectroscopy, Gas Chromatography, Liquid Chromatography, and Flow Cytometry, among others.

Intended Applications

The SpinCon® PAS 450-10 sampler is used to sample biopathogens, particulates, and chemicals suspended in the air. It has been successfully used to find:

Biological Organisms	Particulates, Vapors & Aerosols
Anthrax Spores (Military Grade)	Military Nerve Agents
Viruses and Coliphage viral material	Military Blister Agents
Bacteria and Coliform bacterial material	Psychotic Agents
Spores & fungal allergens	Explosives
Mold, pollen and spores	Pesticides

Concentrated liquid samples are ideal for many detection technologies. Depending upon the sampling time, the particles or compounds of interest in air can be concentrated by several orders of magnitude. Concentrating the constituents of interest is an effective way to overcome sensitivity problems encountered due to the detection limit of the analytical method being used. Therefore concentrations in the air, which would be undetectable using a conventional air sampler, are now easily detected.

The SpinCon® PAS 450-10A biological aerosol sampler is capable of sampling air at a rate of 450 liters per minute and is configured for approximately 10 milliliters of liquid per sample. It operates in a batch mode, collecting a single sample with each use.

Description

The SpinCon® PAS 450-10A sampling system is approximately 18 inches by 8 inches by 15 inches. The system is equipped with wheels and pull handle to enhance portability. The main components of interest are the power supply, timer/button panel, the collector/concentrator (C/C), the air inlet/outlet, and the operating fluid and sample extraction ports.

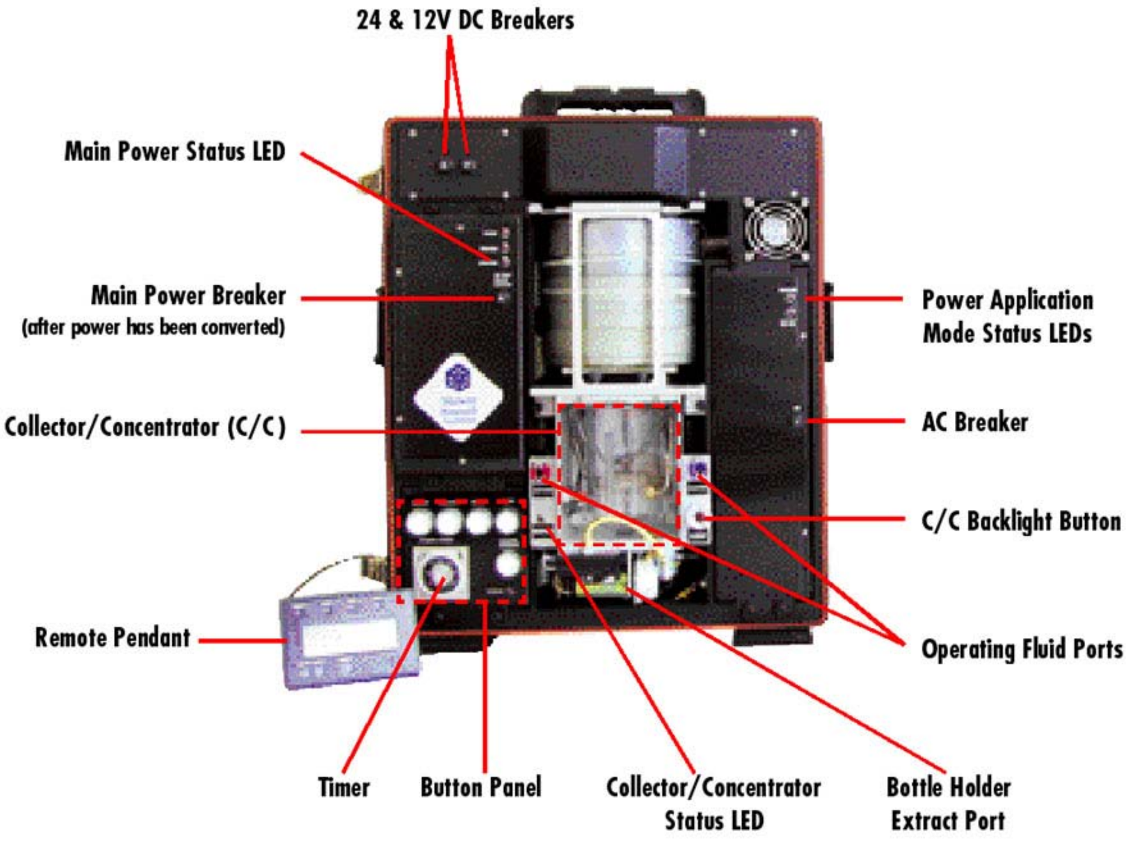

Illustration 1: SpinCon® PAS 450-10A sampler.

Power Supply

The main power switch is located on the button panel. This is a pushbutton that is used to activate powering of the internal components and start collecting sample (as long as power is connected).

The unit can be powered by 120 Volt AC power, or by DC power using either 24 or 12 Volts. A power status LED, located on the upper right of the front panel will indicate which (AC or DC) power source is in use. The unit defaults to AC power when both power supplies are attached.

AC Power

The power cord for 120 Volt AC power is a retractable cord reel accessible on the upper right side of the unit. An AC power breaker is located on the right front of the unit, below the power status LED.

DC Power

The connection terminal for DC power hookup is located on the back of the unit next to the transport handle. Two DC power breakers are located on the upper left corner of the face, one for 24 Volt DC and one for 12 Volt DC.

A backup breaker switch is located on the left front of the unit, just below the DC supply LED status light.

Timer and Button Panel

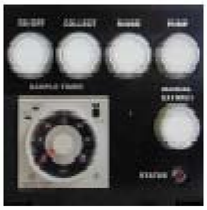

The Timer and Button Panel is located on the bottom left of the front. It consists of an analog timer and five control buttons.

The analog timer is used by manually turning the dial clockwise to set the desired sampling time. The maximum sampling time available is 6 hours. The timer also has several adjustment screws located on the face around the timer dial for changing the resolution of the timing. The user can adjust the timing units (hrs, min, sec, 10h) by adjusting the screw in the lower right, and the timer scale by adjusting the screw in the lower left. A mode indicator is in the upper right of the timer, and must always remain set to mode “A”.

The Button Panel consists of a red status LED, the analog timer, and buttons that control various stages of the sampler operation. Lamps in each of the buttons indicate the current cycle.

Control buttons for the sampler are: “**ON/OFF**”, “**COLLECT**”, “**RINSE**”, “**PUMP**”, and “**MANUAL EXTRACT**”.

Remote Control Panel

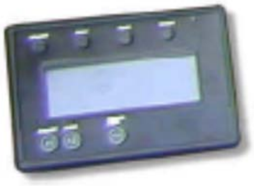

The Remote Control Panel consists of an LCD display, buttons, and LEDs that control and provide information throughout various stages of the sampler operation. When in use, a red LED will illuminate indicating the current cycle, and the LCD interface screen will display status information.

The Remote Control Panel plugs into the left side of the unit. Buttons found on the Remote Pendant sampler are: “**SAMPLE**”, “**RINSE**”, and “**FLUID INJECT**”.

Trigger Port

A trigger port is located directly above the connector for the Remote Control Panel on the left side of the unit. This port allows the user to provide a switch closure to begin or stop a collection cycle. The function of the switch closure mirrors the function of the collect button on the unit.

Collector/Concentrator (C/C)

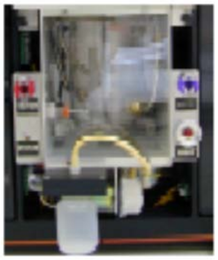

The Collector/Concentrator (C/C) is where the air sample is routed through the unit and the target material concentrated in the liquid phase. As air passes through the C/C, cyclonic mixing transfers a high portion of the target particles or compounds in air to the liquid medium. The liquid medium remains in the C/C as air is sampled to continuously concentrate the constituents of interest into the liquid. The mixing of air with water also causes liquid evaporation during sampling. Evaporated water is replaced by injecting makeup water throughout the sampling process.

A red LED, located just to the left of the C/C should be illuminated when the unit has power applied. After the chosen sampling interval, the liquid in the C/C can be drawn off as a discrete liquid sample. This sample can then be analyzed for the constituents of interest.

Air Inlet/Outlet

The blower provides an airflow of 450 liters per minute by drawing air from the left side of the unit and then through a slit cut into the C/C. After passing through the C/C and blower, scrubbed air is exhausted through the right side of the unit near the top.

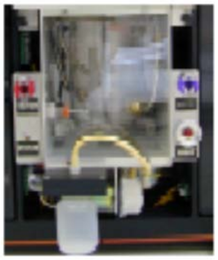

Operating Fluid and Sample Extraction Ports

The Operating Fluid Ports are found on the lower front of the unit. The blue port, on the right side of the C/C, is used for the sterilized water connection. The red port, on the left, is used for the sampling solution.

The SpinCon® PAS 450-10 system is shipped with an initial supply of sampling fluids: a sampling solution for sample collection and sterile water for makeup liquid and rinsing. Additional sampling fluids may be reordered from Innovaprep LLC, 132 E Main St, Drexel MO, 64742. The refrigerated shelf life of the fluids is 2 months (1 month unrefrigerated).

The Sample Extraction Port, or bottle holder, is found on the front of the unit near the bottom. This is where the sample collection bottle is inserted by removing the lid and latching it in place. After sampling is complete, the sample bottle will contain all the fluid that had been in the C/C during sampling. At the end of sampling, the sample bottle is removed by unlatching it from the port. The system uses disposable polyethylene collection bottles. Additional collection bottles may also be reordered from Innovaprep.

LED Status Lights

There are four LED Status Lights located across the front of the unit that indicate if power is correctly applied. All four LED Status Lights should be illuminated when the sampler is turned on.

Specifications

- Power Requirements: 120 VAC or 12/24 VDC
- Power Consumption: approximately 450 W
- Airflow Rate: 450 L/min
- Size: 18 inches x 15 inches x 8 inches
- Weight: 46 pounds
- Sampling Time: Selectable between 5 minutes and 6 hours
- Operating Temperature Range: 2° to 50°C (36° to 120°F)
- Sample Size: 10 milliliters
- Portability: Easily transportable in protective carrying case
- Sample Extraction: Automatic into sample bottle; features bottle presence detection
- Solution: Uses phosphate-buffered saline in combination with sterile water for sample collection.
- Controls: Integral control with LCD status display; sample, rinse, sample time, and manual extract and inject controls
- Decontamination: Exterior surfaces can be wiped with dilute bleach solution. Fluid lines and contactor can be flushed with bleach solution followed by rinses with sterile water.

Section 2: Setup and Use

The SpinCon® PAS 450-10 sampling system operates on 120 Volts AC or 12/24 Volts DC power. It should not be turned off unless all cycles are completed as indicated by the illuminated “ON/OFF” button or by the remote pendant showing “READY”. If power is lost, or the unit is shut down before it is in the “READY” mode, some minor adjustments will need to be made as described in the Shutdown section later in this manual.

The sampling system uses about a 10 milliliters charge of sampling fluid during the initial fluid injection and about 1 to 2 milliliters of sterile water per minute of sampling (depending on environmental conditions). Expect 2mL per minute at high ambient temperatures (>80°F) and low humidities (<15% RH), whereas less than 1-mL per minute evaporation can be expected at high humidities (>80% RH) and low temperatures (<60°F). Additionally, about 12 milliliters of sterile water will be used per rinse cycle. Running a rinse cycle after every sample cycle is highly recommended.

Setup

Connect the unit to an appropriate (120 Volts AC or 12/24 Volts DC) power source and make sure sampling fluids are placed in their proper ports.

Powering On

1. Connect the unit to an appropriate power source.
2. Make sure all breakers are pressed in and push the “ON/OFF” button.
3. The “ON/OFF” button and four red status LEDs will illuminate, indicating that power is applied to all the hardware boxes.

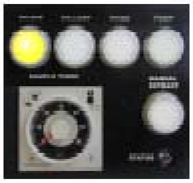

Connecting Fluids

1. Hang the fluids bags on the hooks provided above the ports and connect the bag outlet to the proper port**. Make sure that the correct fluids are connected to their corresponding fluid ports as described in Section 1, and on the system labels**.

2. Place the sample bottle in the sample extraction port (bottle holder) by simply removing the lid, pressing in the locking plate, inserting the bottle, and releasing the locking plate.

Use

Starting Collection Using the Button Panel

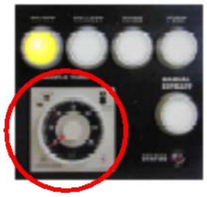

1. Set the sample timer to the desired sampling time by manually turning the timer dial clockwise. The maximum sampling time that should be set is 6 hours.

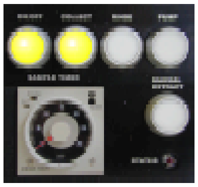

2. Sample collection is started by pressing the “COLLECT” button on the button panel. The SpinCon® sampling system will initiate sampling and the following events will be observed:

   - The “COLLECT” button and the filling LED will illuminate, and the “PUMP” LED will flash.
   - There will be an initial injection of approximately 10 milliliters of the sampling solution. This takes about 45 seconds.
   - After the unit is done filling, the filling LED will turn off, the “PUMP” LED will pulse periodically, and the timer will start as indicated by a green flashing LED on the timer.
   - The unit will periodically inject water to compensate for fluid evaporation, as indicated by the illuminated “PUMP” LED. Monitoring the “PUMP” LED can give a good indication of the current fluid usage, as each flash of the LED indicates a 250µL sterile water injection.

3. At the end of the manually set time, the blower will slow and the unit will pump the sample fluid into the sample bottle. The timer can also be manually turned to “0” minutes to end the sampling cycle.
   Note: If a bottle is not present in the extraction port bottle holder, the unit will wait. The unit will automatically transfer the sample once a bottle is put in place.
   Once the sample collection has completed, press the “COLLECT” button to end the cycle.

4. Pressing the “COLLECT” button returns the unit to its “READY” status and ends the collection cycle. Remove the sample bottle from the bottle holder and replace the lid.

5. If the fluid is not completely extracted at the end of sampling, pressing the “MANUAL EXTRACT” button will turn on the extraction pump. Press the “MANUAL EXTRACT” button again to stop the pump. Beware

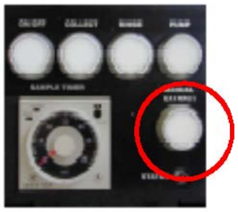

that while in this cycle, the unit will not check for the presence of a collection bottle in the bottle holder before starting

6. Perform a rinse cycle by inserting a rinse collection bottle into the bottle holder and pressing the “RINSE” button. When the “RINSE” button is pressed, the following rinse sequence will automatically occur:

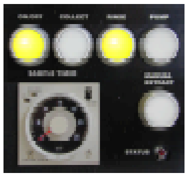

   - The “RINSE” button will illuminate, and the “PUMP” LED will flash.
   - After the initial injection is done, the “PUMP” LED will stop flashing.
   - After a timed rinse period expires, the rinse fluid will be deposited into the waiting bottle.
     - If a bottle is not present in the extraction port bottle holder, the unit will wait. The unit will automatically extract the rinse water once a bottle is put in place.
     - If the rinse fluid is not completely extracted from the sampler, pressing the “MANUAL EXTRACT” button will turn on the extraction pump. Pressing the “MANUAL EXTRACT” button again will turn off the pump

7. After the rinse cycle has completed, press the “RINSE” button again to end. Pressing the “RINSE” button once again completes the rinse cycle and returns the unit to the “READY” mode.

Starting Collection Using the Remote Control Panel

1. Set the sample timer to the desired sampling time by manually turning the time dial clockwise. The maximum sampling time that should be set is 6 hours.

2. Sample collection is started by pressing the “SAMPLE” button on the keypad found beneath the pendant LCD screen. The SpinCon® sampling system will initiate sampling and the following events will be observed:

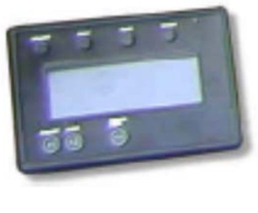

   - The “SAMPLE” LED will illuminate, the “PUMP” LED will flash, and the interface screen initially displays “Collection Cycle, charging.”
   - There will be an initial injection of approximately 10 milliliters of the sampling solution. This takes about 45 seconds.

- After the unit is done charging the "PUMP" LED will pulse periodically and the timer will start. The screen will display "Collection Cycle", while showing elapsed sampling time in seconds.
- The unit will periodically inject water to compensate for fluid evaporation, as indicated by the "PUMP" LED.

3. At the end of the manually set time, the screen will display "Transferring Sample." The blower will slow and the unit will pump the sample fluid into the sample bottle. The timer can also be manually turned to "0" minutes to end the sampling cycle.
   Note: If a bottle is not present in the extraction port bottle holder, the unit will wait and the pendant will display "Waiting for Bottle." The unit will automatically transfer the sample once a bottle is put in place.
   Once the sample collection has completed, the LCD screen displays "Transfer Complete, Press 'SAMPLE' to end."

4. Visually verify that the entire sample has been transferred, and then press the "SAMPLE" button. This returns the unit to its "READY" status, as displayed on the screen, and ends the collection cycle. Remove the sample bottle from the bottle holder and replace the lid.

5. If the fluid is not completely extracted at the end of sampling, pressing the "MANUAL EXTRACT" button will turn on the extraction pump. Press the "MANUAL EXTRACT" button again to stop the pump. Beware that while in this cycle, the unit will not check for the presence of a collection bottle in the bottle holder before starting

6. Perform a rinse cycle by inserting a rinse collection bottle into the bottle holder and pressing the "RINSE" button. When the "RINSE" button is pressed, the following rinse sequence will automatically occur:

   - The "RINSE" LED will illuminate, the "PUMP" LED will flash, and the interface screen initially displays "Rinse Cycle, Injecting rinse"
   - After the initial injection is done, the "PUMP" LED will stop flashing and the interface screen displays "Rinse Cycle, Rinsing Contactor/Time Remaining" while showing the remaining time.
   - After the count down on the rinse cycle is complete, the interface screen displays "Rinse Cycle, Transferring Rinse". The rinse fluid will be deposited into the waiting bottle.
     - If a bottle is not present in the extraction port bottle holder, the unit will wait and the pendant will display "Waiting for Bottle". The unit will automatically extract the rinse water once a bottle is put in place.

- If the rinse fluid is not completely extracted from the sampler, pressing the "MANUAL EXTRACT" button will turn on the extraction pump. Pressing the "MANUAL EXTRACT" button again will turn off the pump

7. After the rinse cycle has completed, the interface screen displays "Transfer Complete, Press 'RINSE' to end". Pressing the "RINSE" button once again completes the rinse cycle and returns the unit to the "READY" mode, which will be displayed on the LCD screen.

Manual Functions

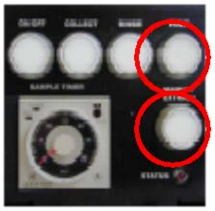

The "MANUAL EXTRACT" and "PUMP" control buttons are used as backup options.

Manual Extraction

As also discussed in Steps 5 and 6, above, fluid can be manually extracted from the C/C. If the sample or rinse fluid is not completely extracted, or for some other reason you want to collect the fluid in the C/C, use this option. Pressing the "MANUAL EXTRACT" button will turn on the extraction pump.

Note that the "MANUAL EXTRACT" cycle will not check for the presence of a collection bottle so this needs to be in place first.

Pressing the "MANUAL EXTRACT" button again will turn off the pump.

Manual Fluid Injection

The "PUMP" is used to add more fluid to the C/C, but should not be needed during normal use. Pressing the "PUMP" button will inject one 250-microliter pulse of water into the C/C. This button can also be used to add extra fluid during a rinse cycle which is useful to ensure that the fluid level rises high enough in the C/C to rinse off dirt above the collection water line, or to ensure that any bleach in the contactor is removed after a bleach rinse.

Shutdown

Remove the sampling and rinse fluids from the Fluid Injection ports. Make sure that the "COLLECT", "RINSE", "PUMP", and "MANUAL EXTRACT" buttons are not illuminated. Then push the "ON/OFF" button. Unplug the unit from its power source. The unit is ready for whatever care and cleaning are needed. For information on care and cleaning, see the following Section 3.

Unintended or Premature Shutdown

If power is lost or the unit is turned off without ending a system cycle (i.e., “COLLECT” or “RINSE” buttons are illuminated on the Button Panel, or not in the “READY” mode on the Remote Control LCD panel), the unit will resume from the same state it was in at shutdown once power is turned back on. The elapsed time will read 00000; however, the manual timer will reset to its full value and the sample will continue for the full duration listed on the timer.

If you do not wish to resume the cycle at this point, press the appropriate use button (e.g., the “SAMPLE” button or the “RINSE” button) to return the unit to the “READY” mode. Cycle the power off, then on again to re-initialize the system.

Section 3: Care

WARNING: Before any maintenance action, unplug AC or DC power supplies.

Operator Maintenance

Maintenance involves weekly surface cleaning, checking for loose connections, decontamination and rinsing of the collector, cleaning around the collector, and cleaning of the air passage ways. Cleaning around the contactor should be performed if dust or oil is visible between the contactor and photoeyes (the photoeyes are mounted on the "C" bracket surrounding the contactor). To clean around the contactor, requires removing the clear polycarbonate window using the four attaching screws. Cleaning of the contactor should always be done using an oil free cloth or paper towel. Water or rubbing alcohol can be used to aid in the removal of any dirt or dust from the contactor. Contactor cleaning is discussed further in later sections.

Minor repairs and parts replacements should be advised by the technical support team. Replacing components, recalibration and major repairs are performed by Innovaprep.

Cleaning External Components

The unit can be wiped down using a damp cloth with a 10% bleach-water or mild cleaning solution.

Cleaning Internal Fluid Lines

After use, both lines should be flushed with a 10% bleach-water solution, followed by several water rinses. Flushing is necessary to help minimize biological growth in the fluid lines. The unit should be flushed if it will not be used for more than a day.

Flush Procedure

Connect a source of bleach-water solution to each fluid port, set the sample time to "0" minutes, and press the "SAMPLE" button. This will flush the fluid lines from the sample fluid port to the C/C. To flush the fluid lines from the rinse fluid port to the C/C press the "RINSE" button after the zero duration sample cycle has completed. Wait for 5 minutes before continuing with any follow-up rinses in order to allow the bleach to act. Then rinse the bleach from both lines of the unit by repeating these steps using sterile water. NOTE: Allowing bleach to remain in

the lines for too long can reduce the lifetime of the system's fluid injection pump and cause sample degradation.

Alternatively, if only one source of bleach-water solution is available, a collect and rinse may be run in succession by connecting the source to the left port and running a short collection, and then placing the source on the right most port and running a rinse. Ensure that both ports are rinsed thoroughly with water and that several collections and rinses are run with water to remove any residual bleach.

As an option an additional rinse could be performed using a sodium thiosulfate solution after the bleach rinse and before the water rinses. This may be useful for prolonging the life of the internal components that had been in contact with the corrosive bleach solution, as well as providing extra certainty that residual bleach will not affect any subsequent samples.

Rinse both lines several times with sterilized water after performing a sodium thiosulfate rinse..

If the unit has been unused for a period of time it is recommended that the same flush procedure be performed prior to use. This is recommended in case any growth has occurred in the lines.

Note:

As noted in the "Use" section of this manual, the unit should always be put through a "RINSE" cycle after every sample collection. This is considered part of the normal operation of the unit and is included here to stress its importance.

Contactor Cleaning

Note: It is critical that the contactor glass in front of the level sensors stays clean. An indicator that the contactor should be cleaned is if the operator begins to notice that sample volumes are significantly more or less than 10 ml. Also, visible coatings on the interior or exterior of the contactor should be cleaned periodically before it becomes a larger problem.

Cleaning Exterior of Contactor

STEP 1: Remove four screws from plexi-glass covering contactor box.

STEP 2: Carefully clean exterior of glass contactor with a cloth dampened with water or alcohol. Pay particular attention to not disturb the positioning of the contactor, injector nozzle, or level sensors. Always use an oil free cloth or paper towel for cleaning the contactor.

STEP 3: Most importantly, ensure that glass in front of level sensors is cleaned.

Cleaning Interior of Contactor

STEP 1: Remove contactor/blower module

– Unscrew two knurled fasteners at the sides of the blower mounting plate

– Unscrew inlet and outlet flanges from the sides of the outside of the case, being careful to apply pressure to the front of the blower module to prevent binding. The inlet and outlet, once initially loosened, should be freely rotated by finger pressure. If not, binding is occurring, and correct pressure on the blower module is not being applied. Check for clearance between the flange being removed and the system case, and lessen or increase pressure as needed to allow clearance. If excessive binding occurs, the connection threads may gall, potentially resulting in seizing of the inlet or outlet flanges to the module and damaging the system blower.

– Once the inlet and outlet tubes are removed, slide the contactor/blower module forward and out of the case. If necessary, unplug the wiring harness from the green 12-pin receptacle on the contactor box to allow additional flexibility.

STEP 2: Unscrew four knurled fasteners on top of blower plate. There are two knurled fasteners on the front of the module, and two on the back.

STEP 3: Separate the contactor box from the blower. DO NOT remove the four flathead screws securing the contactor to the box.

STEP 4: Carefully clean interior of glass with a cloth dampened with water or alcohol. Always use an oil free cloth or paper towel for cleaning the contactor.

STEP 5: Most importantly, ensure that glass in front of level sensors is cleaned.

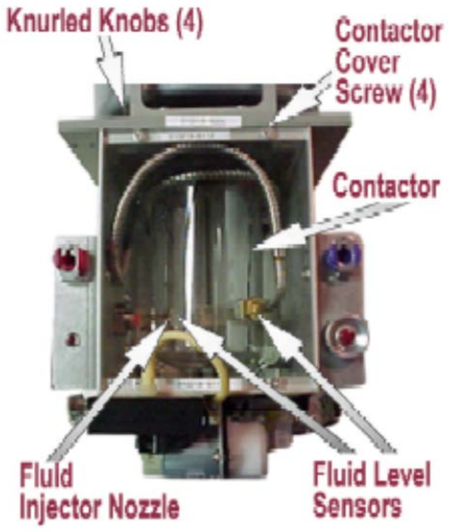

Illustration 2: SpinCon PAS450-10A Contactor Box.

Supplies

The refrigerated shelf life of sampling fluids is two months. It is recommended that sampling fluids be kept refrigerated.

Yearly Preventive Maintenance

The unit should be sent in annually for preventative maintenance. First, clean the external components and internal lines with a 10% bleach solution as previously described in Section 2.

Contact Innovaprep at 816-619-3375 for a Return Materials Authorization (RMA) and shipping instructions.

If the unit has collected infectious material, you must notify Innovaprep of this when requesting the RMA. A certificate of decontamination will be required prior to shipping the unit.

Section 5: Decontamination

The Field Decontamination Procedure is:

Step 1: Remove the sampler from the "hot zone" to an area where decontamination can be safely performed.
Step 2: Wipe the exterior of the case, air inlets, handles, water/PBS bags, external hoses and platform around the sampler with 10% bleach solution.
Step 3: Run the Decontamination Fluid (10% bleach solution) through the collect and one rinse cycle. Cover the intake port with bleach soaked wipe during cycles. Allow to stand for 10 minutes.
Step 4: Conduct two rinse cycles with sterile water following a 5 minute wait time.
Step 5: Conduct third rinse cycle with sterile water and collect sample for testing.
Step 6: Run an analysis on the rinse water sample to determine status of decontamination.

NOTE: These procedures may vary according to the specific situation in which collections are being made, or tailored to a specific pathogen of interest. For certain pathogens, the SpinCon needs to be double bagged and a more complete gas decontamination of the system should be preformed using Formaldehyde or Hydrogen Peroxide in an approved gas chamber.

Users may coordinate with Innovaprep to provide a SpinCon loaner during the decontamination process if required. There will be a fee for the loaner and availability of loaners at times may be limited due to unforeseen circumstances.

A Decontamination Decision Tree is provided to assist the user. Recommended is to treat all verified hot zones as needing a complete gas decontamination of the system to minimize the possibility of internal contamination.

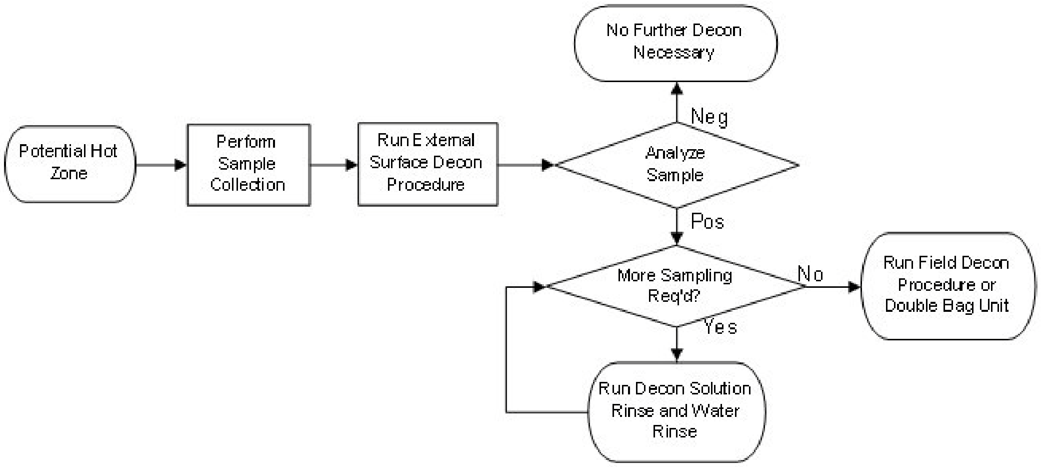

Section 5: Environmental Limitations

Keep in a dry environment. The unit is not rainproof or waterproof

Operate in a low dust environment. Excessive dust can cause premature wear and could cause fluid level control to fail over time without proper preventive maintenance.

The operating temperature range is 36° to 120°F (2° to 50°C).

The operating humidity range is 0 to 100% Relative Humidity (non-condensing).

Chemical compounds used for decontamination of the unit may limit the life of some components. If the unit has been decontaminated after collecting infectious material, it should be sent to Innovaprep for preventative maintenance immediately thereafter.

Section 6: Safety Precautions

This unit is not a closed system. Some air leakage from the collection chamber may occur.

Use caution and treat all collected samples as if they actually contain the materials for which they are being sampled.

Be aware that the unit should be electrically grounded and not used in situations where electrical shock is likely (do not place or operate the unit in standing water).

Warnings:

DO NOT operate the unit with the covers removed.

DO NOT operate the unit in the rain.

DO NOT place, or operate the unit in standing water.

DO NOT soak the unit when cleaning, wipe with a damp cloth containing cleaning solution, water, or alcohol only.

DO NOT restrict the inlet or outlet during operation, as this will reduce performance.

DO NOT drop the unit as it can damage the electronics and the glass C/C.

Section 7: Troubleshooting

Troubleshooting information for the SpinCon® bioaerosol sampler is listed below. In most cases when trouble is encountered, a review of the operating instructions will provide a solution.

Symptom	Cause	Remedy
The unit does not come on when plugged in and on/off button is pressed.	The system electrical breakers may not be activated.	Make sure all breakers on the unit are pushed in and the “ON/OFF” button has been pressed.
	Module connectors may be loose	Check that all green wiring harness connectors are firmly seated.
During sampling, Elapsed Timer does not count.	Unit was shut down without ending a cycle.	Re-initialize unit by turning off the “RINSE” and “SAMPLE” cycles and the turning power off then back on.
Certain functions do not seem to work properly when buttons are pressed	Module connectors may be loose	Check that all green wiring harness connectors are firmly seated.
Fluid does not extract after sampling is complete	Clogged fluid extract line	Inspect and remove debris from fluid extract fittings or tubing between contactor and extract pump.
	Sample bottle not present	Insert sample bottle into bottle holder.
	Defective bottle presence sensor	Lights should be lit on bottle sensor, and should flash red when an object passes within half an inch of the face of the sensor. If no lights are illuminated, or do not flash, contact Innovaprep for repair.
	Defective extract pump	Contact Innovaprep for repair.
Sample fluid does not inject into contactor	Fluids may not be connected properly, or fluid bags are empty	Fluids must be connected to BOTH the red and blue fluid connectors. Check fluids, referencing the manual for proper connection if needed.
	Clogged or leaking fluid lines	Inspect fluid lines for clogs and clear if necessary. If lines are leaking, check to make sure fittings are tight.
	Defective fluid injection pump	When rinse cycle is activated, a faint clicking noise, approximately twice per second should be heard, if no clicking is audible, contact Innovaprep for repair.
Sample volumes low	Dirty contactor	Clean contactor interior and exterior per preventive maintenance procedures.
Sample volumes high	Blockage in air path	Check for obstructions in air flow path, both inlet and outlet and clear.
	Dirty contactor	Clean contactor interior and exterior per preventive maintenance procedures.
	Blower not tightly connected to contactor box	Check to make sure blower is securely attached to the contactor box.

Section 8: Contact Information

Innovaprep LLC
132 E. Main St
Drexel, Missouri 64114

Customer Service For supplies and service

Phone: 816-619-3375
E-mail info@innovaprep.com

Website http://www.innovaprep.com/

Section 9: Quick Operating Instructions

1. **Plug the retractable cord into an 110 volt AC outlet or establish power with 12 or 24 volt batteries. Cooling fan will turn on if using AC power.**

2. **Decide on method of controlling operation from either the remote unit and/or from the switches on the front of the panel.**

3. **Install Collection Fluid and Sterile Water bags to hooks on black panel above blower motor; connect male connectors to appropriate connector on sampler. The connectors are properly installed when they "click" and are firmly seated. The connectors and bags can be removed after operations are complete by depressing spring on top of female connector and pulling out the male connectors.**
4. **Place sample bottle in holder. Push in slide on left side of bottle holder. Insert bottle without cap. Release slide.**

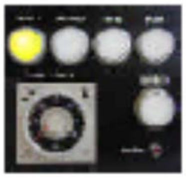

5. **Press ON/OFF button. When on, button lamp will illuminate, secondary cooling fan will activate.**

6. **Confirm that all four status lights are illuminated – one on each module. When using remote unit, the screen message will be "Ready"**

7. **Set the timer to the desired collection time.**

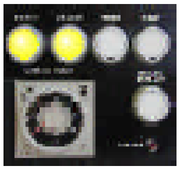

8. **Push the "COLLECT" button on either the air sampler or the remote unit (F1).**

9. **Following the collection cycle, after the liquid has been pumped from the contactor, remove the bottle by pressing the release spring and replace the cap. Place another bottle in the holder.**

10. **Press the "COLLECT" button, on either the air sampler or the remote unit (F1) to end the collect cycle.**

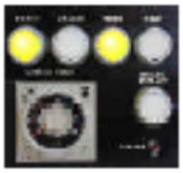

11. **If a rinse is desired or required, depress "RINSE" button on either remote unit (F2) or sampler control panel. Action is confirmed by a button lamp. Since water is being pumped into contactor, only the pump lamp will pulse. Contactor will fill and drain again. Push "RINSE" button off, remove bottle, and dispose of waste. Replace with new sample bottle.**

12. **Be sure that last operation before shut down was a "RINSE"; if not, go to STEP 11. Depress the "ON/OFF" button to shut down; light will extinguish**
