# Supplementary material for: Airborne transmission pathway for coastal water pollution
Source: PeerJ. 2021 Jun 7;9:e11358. doi: 10.7717/peerj.11358 (PMC8191489; doi:10.7717/peerj.11358)
Supplement: Supplemental Information 4 — Like Figure 2, for all aerosol samples with data available (day samples). Right: Average sea surface dye concentrations during the period each aerosol sample was collected at the location on land indicated by the triangle. The 200 m × 100 m dotted box indicates where the average wind direction during each sampling interval intersected the coastal waters from the sampling site. Figure 3 finds the location of that box and its mean dye value for each hourly sea surface dye measurement. Here we show the mean dye field during each air sampling period and the mean upwind location (box). Left: wind rose for the winds observed at the KNRS meteorological station during the aerosol sampling periods. [file peerj-09-11358-s004.pdf]

Figure S3. Mean sea surface dye concentrations upwind of aerosol sampling locations. Like Figure 2, for all aerosol samples with data available (day samples). Right: Average sea surface dye concentrations during the period each aerosol sample was collected at the location on land indicated by the triangle. The 200 m x 100 m dotted box indicates where the average wind direction during each sampling interval intersected the coastal waters from the sampling site. Figure 3 finds the location of that box and its mean dye value for each hourly sea surface dye measurement. Here we show the mean dye field during each air sampling period and the mean upwind location (box). Left: wind rose for the winds observed at the KNRS meteorological station during the aerosol sampling periods.

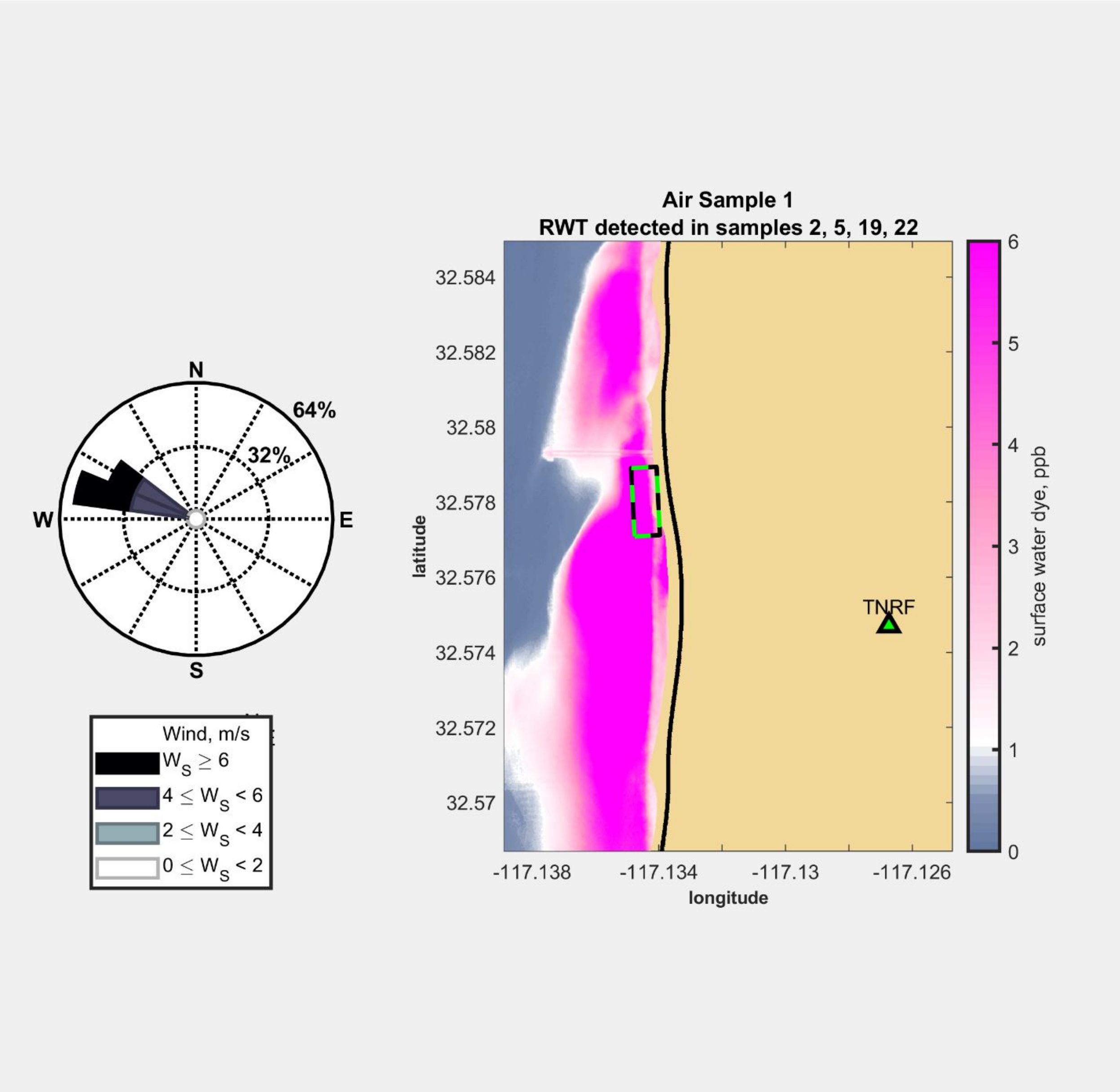

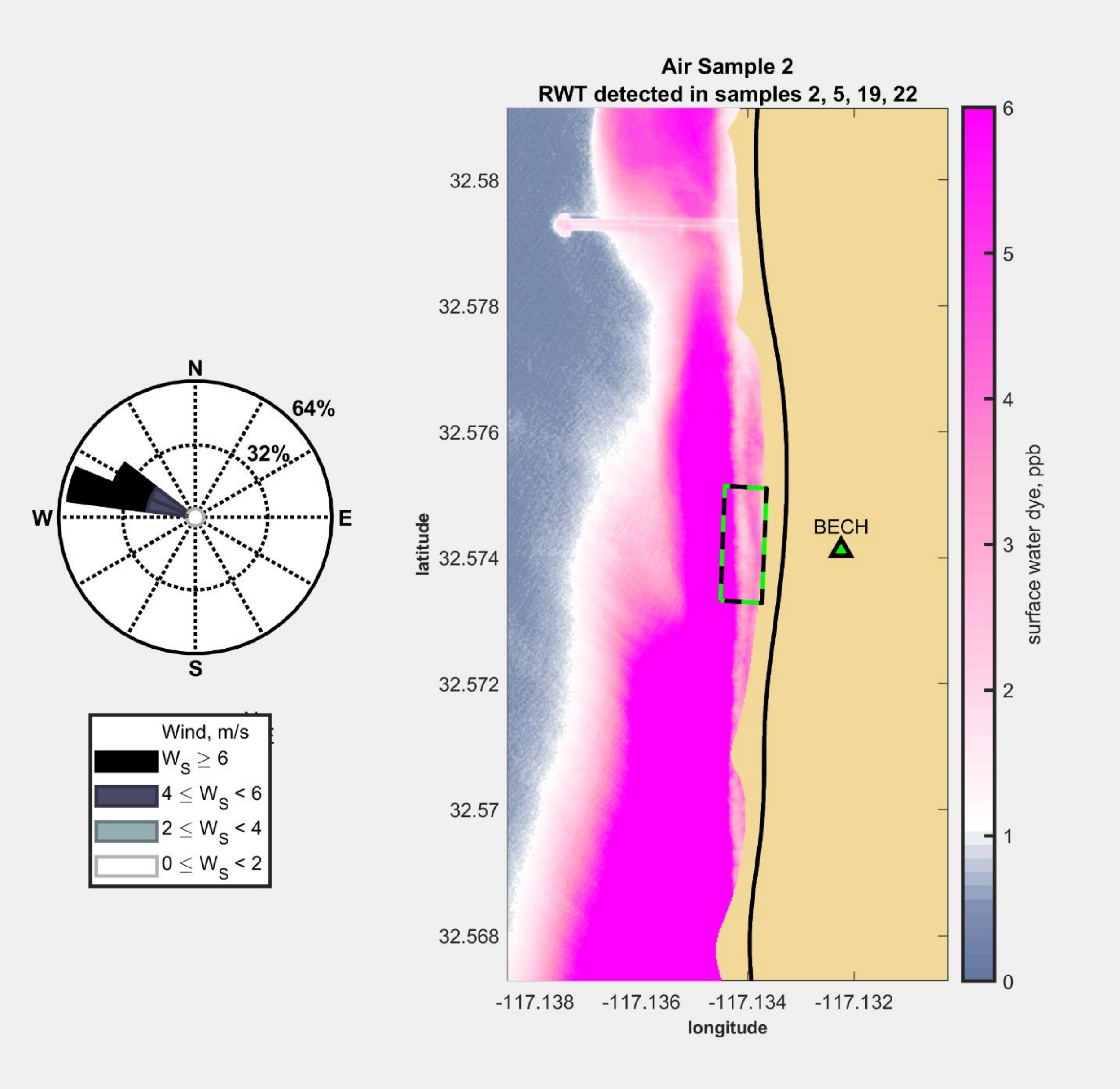

Air Sample 2
RWT detected in samples 2, 5, 19, 22
N
S
E
W
64%
32%
Wind, m/s
$W_S \geq 6$
$4 \leq W_S < 6$
$2 \leq W_S < 4$
$0 \leq W_S < 2$
BECH
latitude
longitude
surface water dye, ppb
32.58
32.578
32.576
32.574
32.572
32.57
32.568
-117.138
-117.136
-117.134
-117.132
0
1
2
3
4
5
6

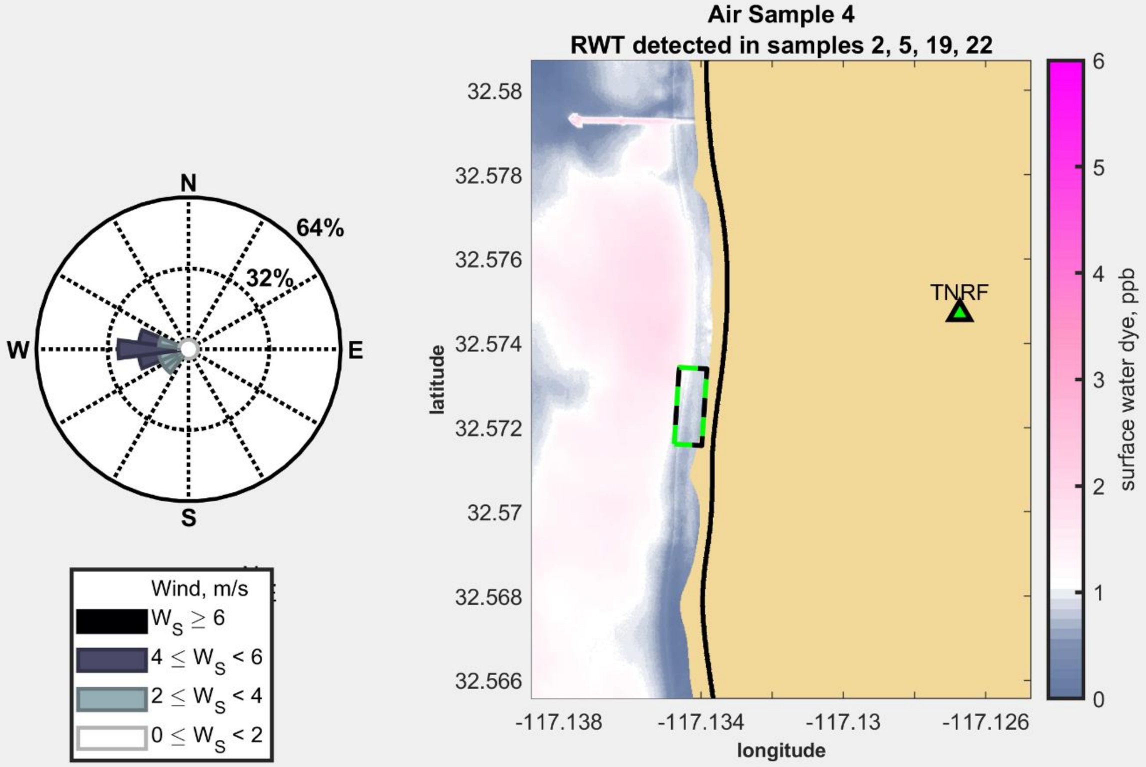

Air Sample 4
RWT detected in samples 2, 5, 19, 22
N
S
W
E
64%
32%
Wind, m/s
$W_S \geq 6$
$4 \leq W_S < 6$
$2 \leq W_S < 4$
$0 \leq W_S < 2$
TNRF
32.58
32.578
32.576
32.574
32.572
32.57
32.568
32.566
latitude
-117.138
-117.134
-117.13
-117.126
longitude
0
1
2
3
4
5
6
surface water dye, ppb

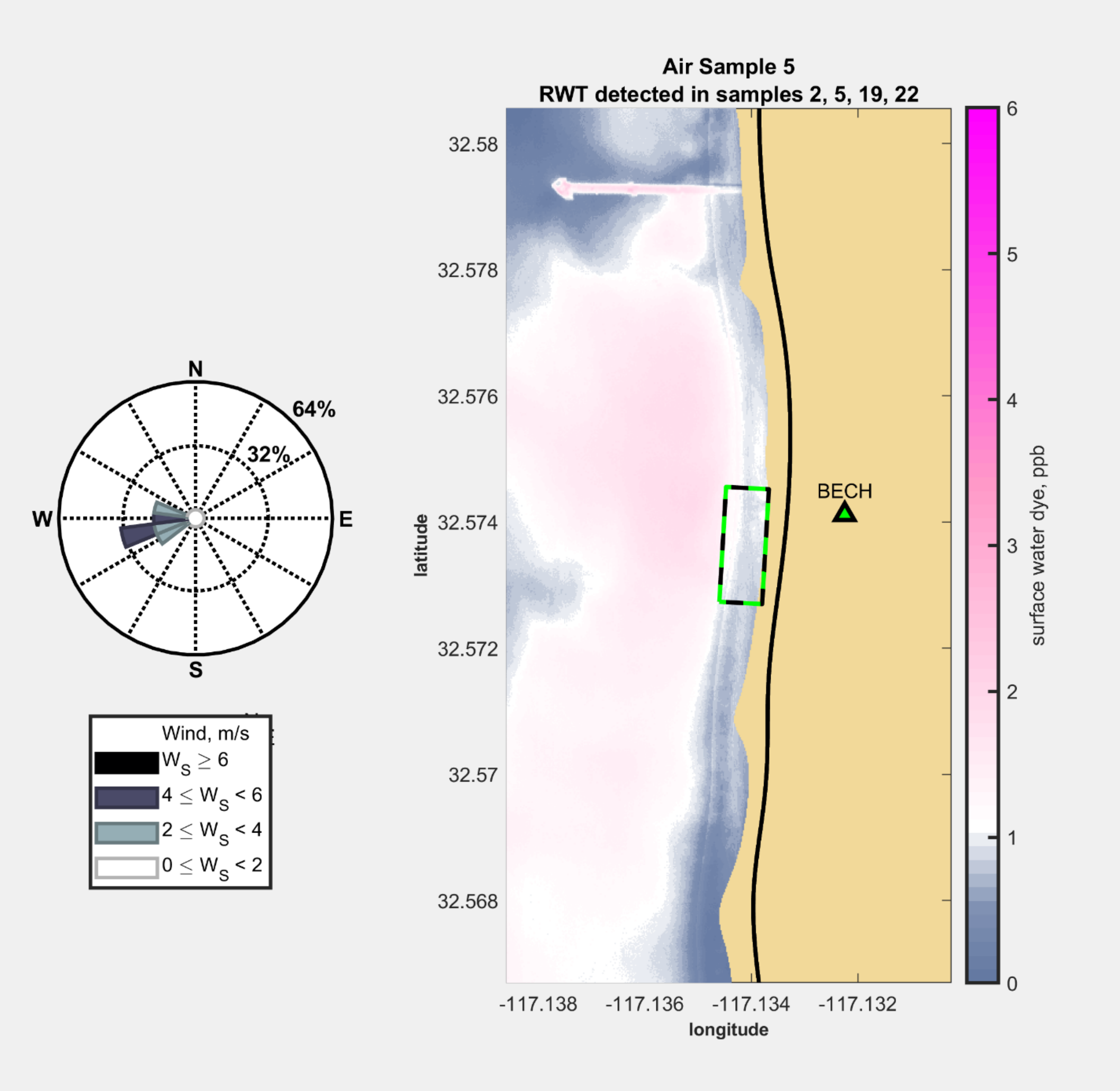
N
64%
32%
W
E
S
Wind, m/s
$W_S \geq 6$
$4 \leq W_S < 6$
$2 \leq W_S < 4$
$0 \leq W_S < 2$
Air Sample 5
RWT detected in samples 2, 5, 19, 22
32.58
32.578
32.576
32.574
32.572
32.57
32.568
latitude
BECH
-117.138
-117.136
-117.134
-117.132
longitude
6
5
4
3
2
1
0
surface water dye, ppb

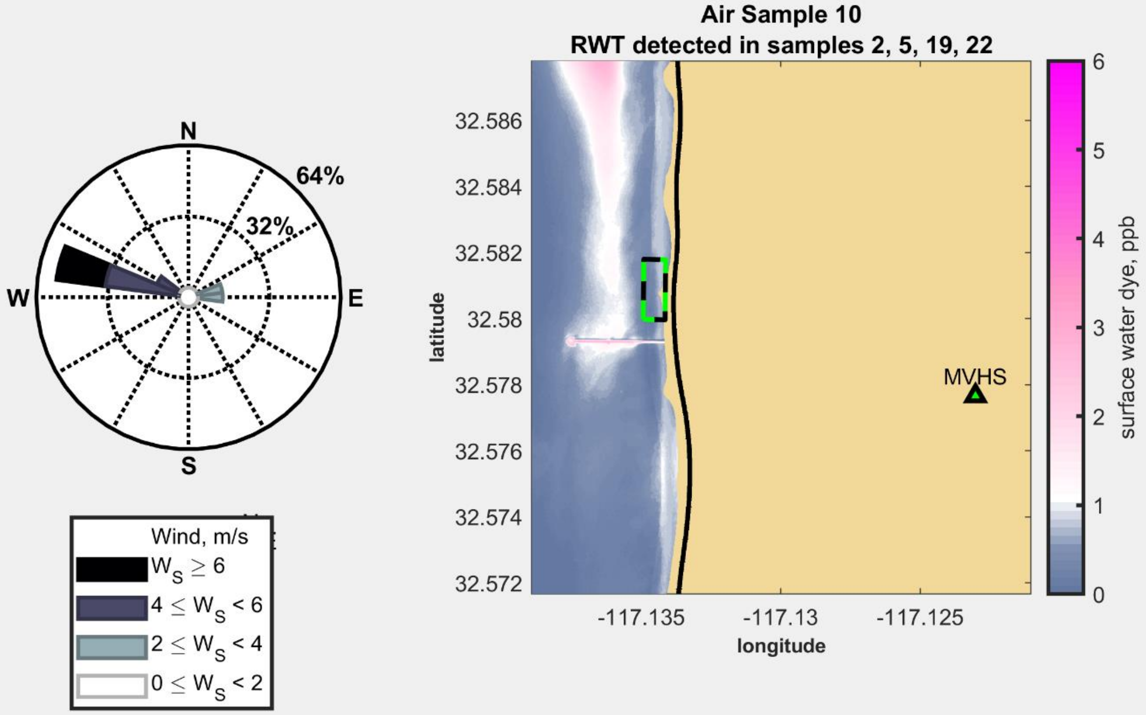

N
64%
32%
W
E
S
Wind, m/s
W_S ≥ 6
4 ≤ W_S < 6
2 ≤ W_S < 4
0 ≤ W_S < 2
Air Sample 10
RWT detected in samples 2, 5, 19, 22
32.586
32.584
32.582
32.58
32.578
32.576
32.574
32.572
latitude
-117.135
-117.13
-117.125
longitude
MVHS
6
5
4
3
2
1
0
surface water dye, ppb

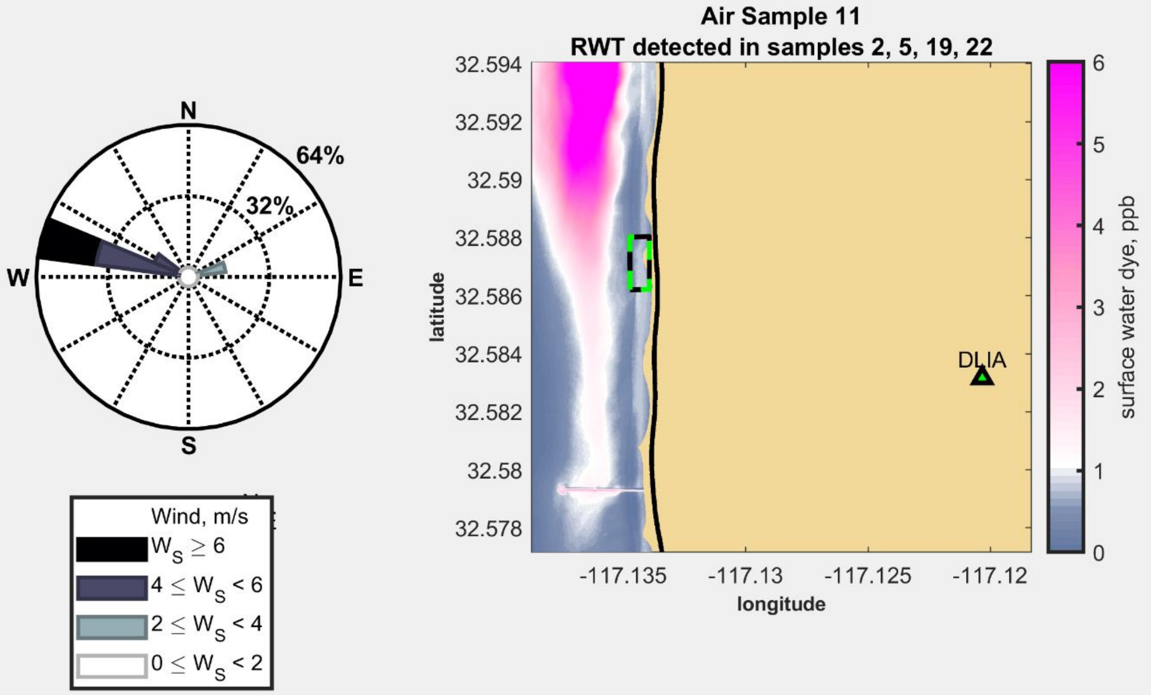

N
E
S
W
64%
32%
Wind, m/s
W_S ≥ 6
4 ≤ W_S < 6
2 ≤ W_S < 4
0 ≤ W_S < 2
Air Sample 11
RWT detected in samples 2, 5, 19, 22
latitude
32.594
32.592
32.59
32.588
32.586
32.584
32.582
32.58
32.578
longitude
-117.135
-117.13
-117.125
-117.12
DLIA
surface water dye, ppb
0
1
2
3
4
5
6

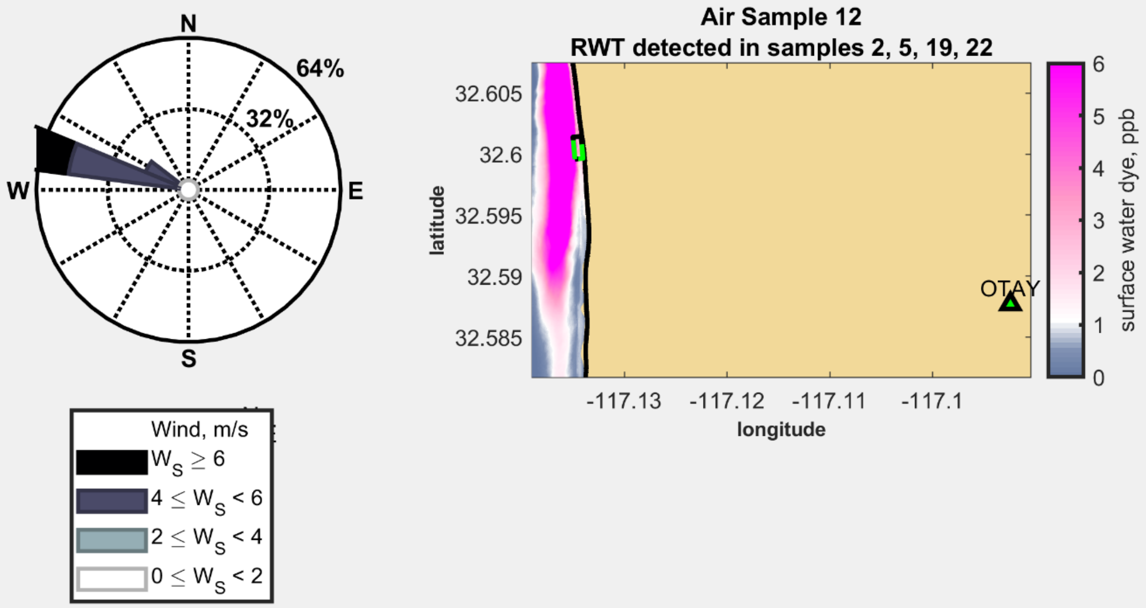

N
64%
32%
W
E
S
Wind, m/s
$W_S \geq 6$
$4 \leq W_S < 6$
$2 \leq W_S < 4$
$0 \leq W_S < 2$
Air Sample 12
RWT detected in samples 2, 5, 19, 22
latitude
32.605
32.6
32.595
32.59
32.585
-117.13
-117.12
-117.11
-117.1
longitude
OTAY
surface water dye, ppb
6
5
4
3
2
1
0

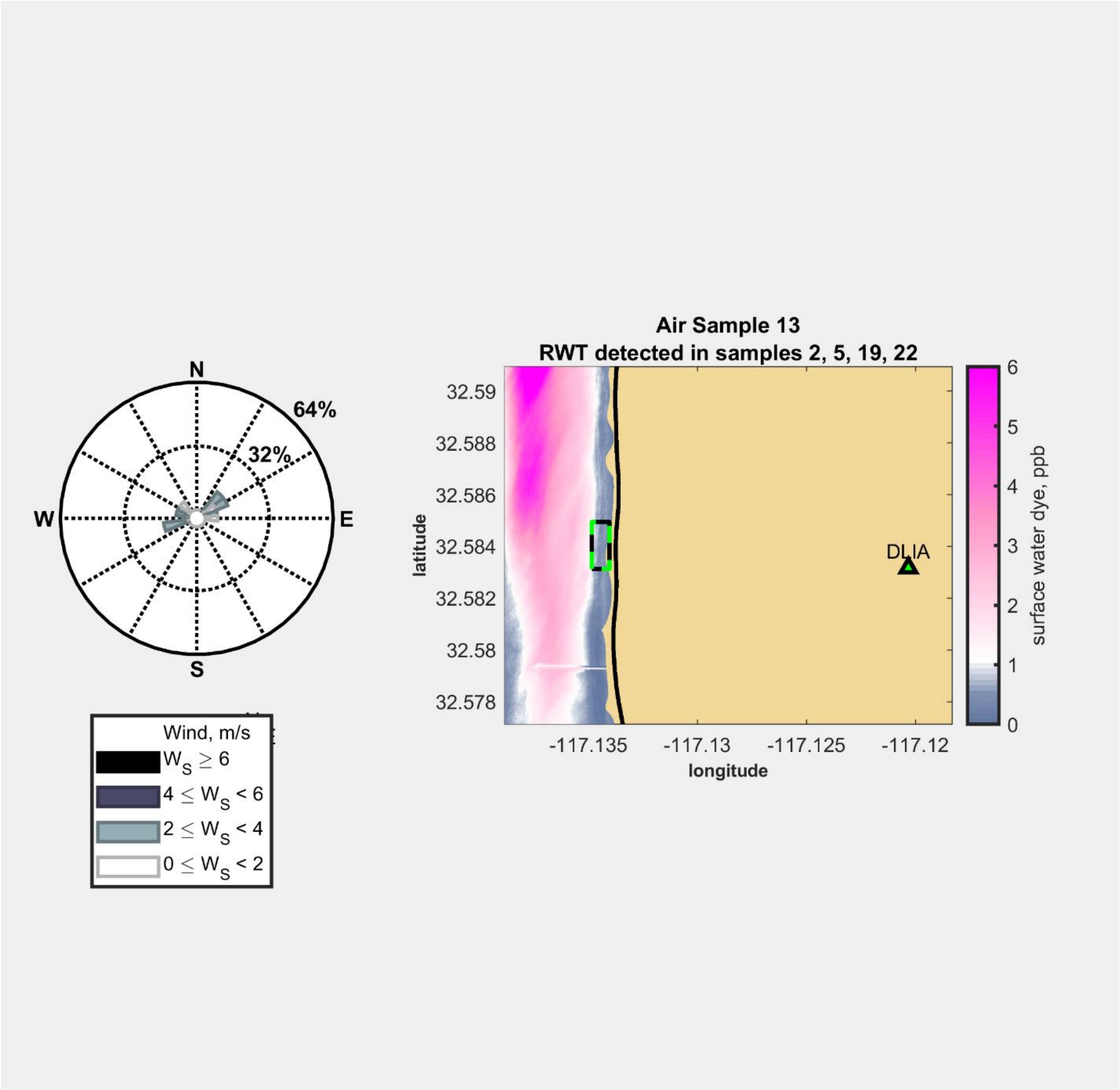
N
E
S
W
64%
32%
Wind, m/s
$W_S \geq 6$
$4 \leq W_S < 6$
$2 \leq W_S < 4$
$0 \leq W_S < 2$
Air Sample 13
RWT detected in samples 2, 5, 19, 22
latitude
32.59
32.588
32.586
32.584
32.582
32.58
32.578
longitude
-117.135
-117.13
-117.125
-117.12
DLIA
surface water dye, ppb
0
1
2
3
4
5
6

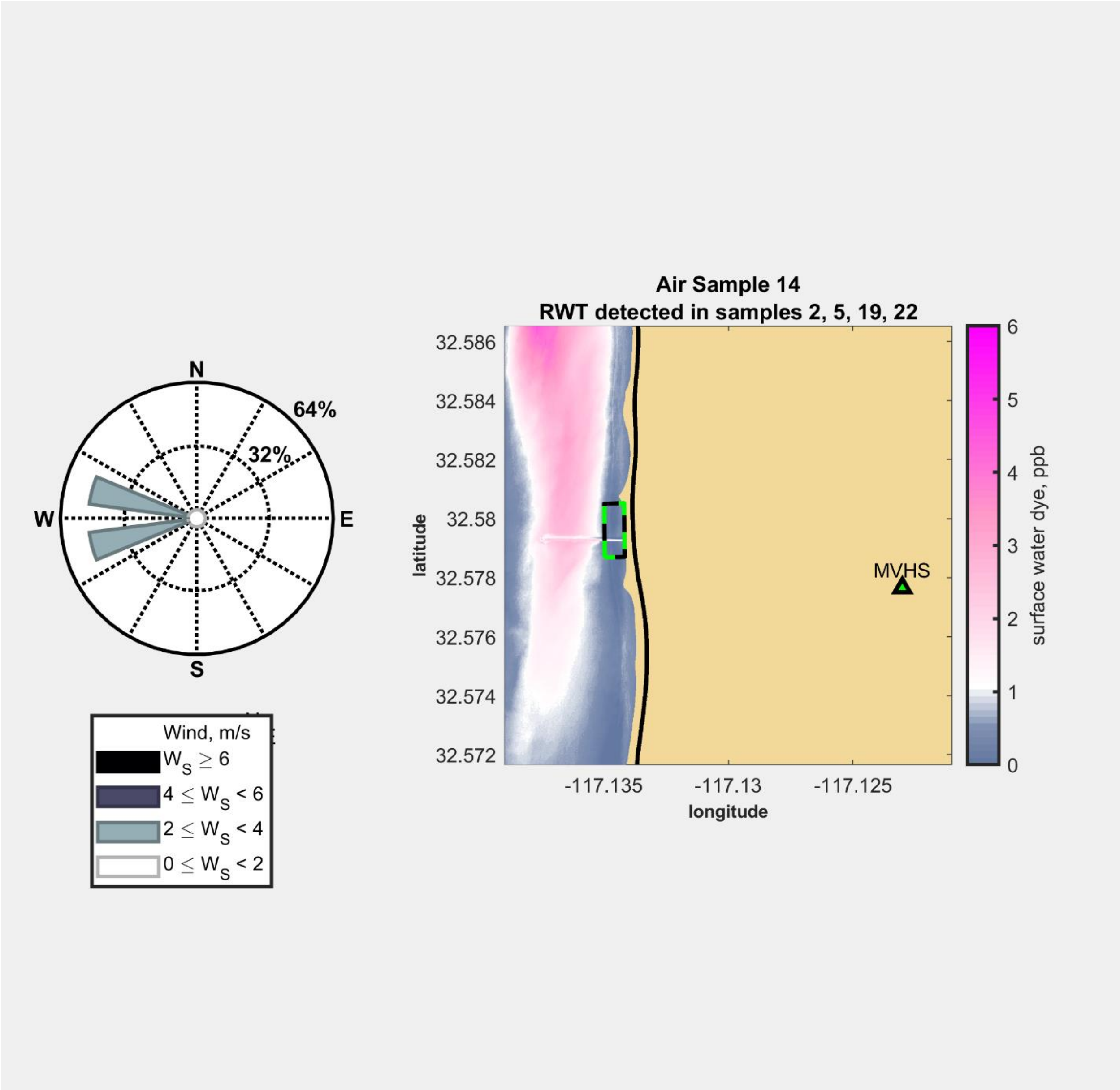

N
S
E
W
64%
32%
Wind, m/s
W_S ≥ 6
4 ≤ W_S < 6
2 ≤ W_S < 4
0 ≤ W_S < 2
Air Sample 14
RWT detected in samples 2, 5, 19, 22
32.586
32.584
32.582
32.58
32.578
32.576
32.574
32.572
latitude
-117.135
-117.13
-117.125
longitude
MVHS
surface water dye, ppb
0
1
2
3
4
5
6

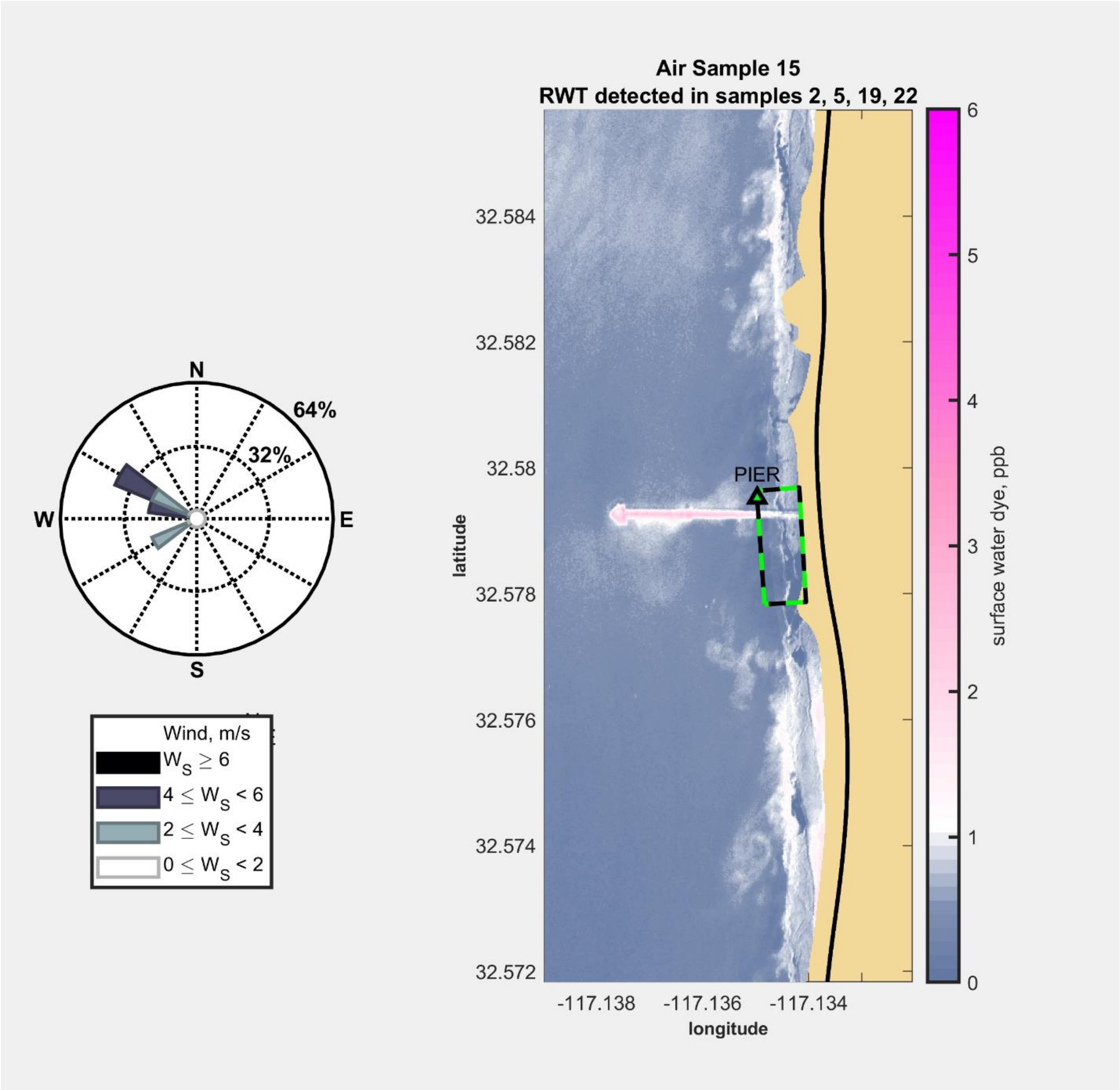

Air Sample 15
RWT detected in samples 2, 5, 19, 22
N
S
W
E
64%
32%
Wind, m/s
$W_S \geq 6$
$4 \leq W_S < 6$
$2 \leq W_S < 4$
$0 \leq W_S < 2$
PIER
latitude
longitude
32.584
32.582
32.58
32.578
32.576
32.574
32.572
-117.138
-117.136
-117.134
surface water dye, ppb
6
5
4
3
2
1
0

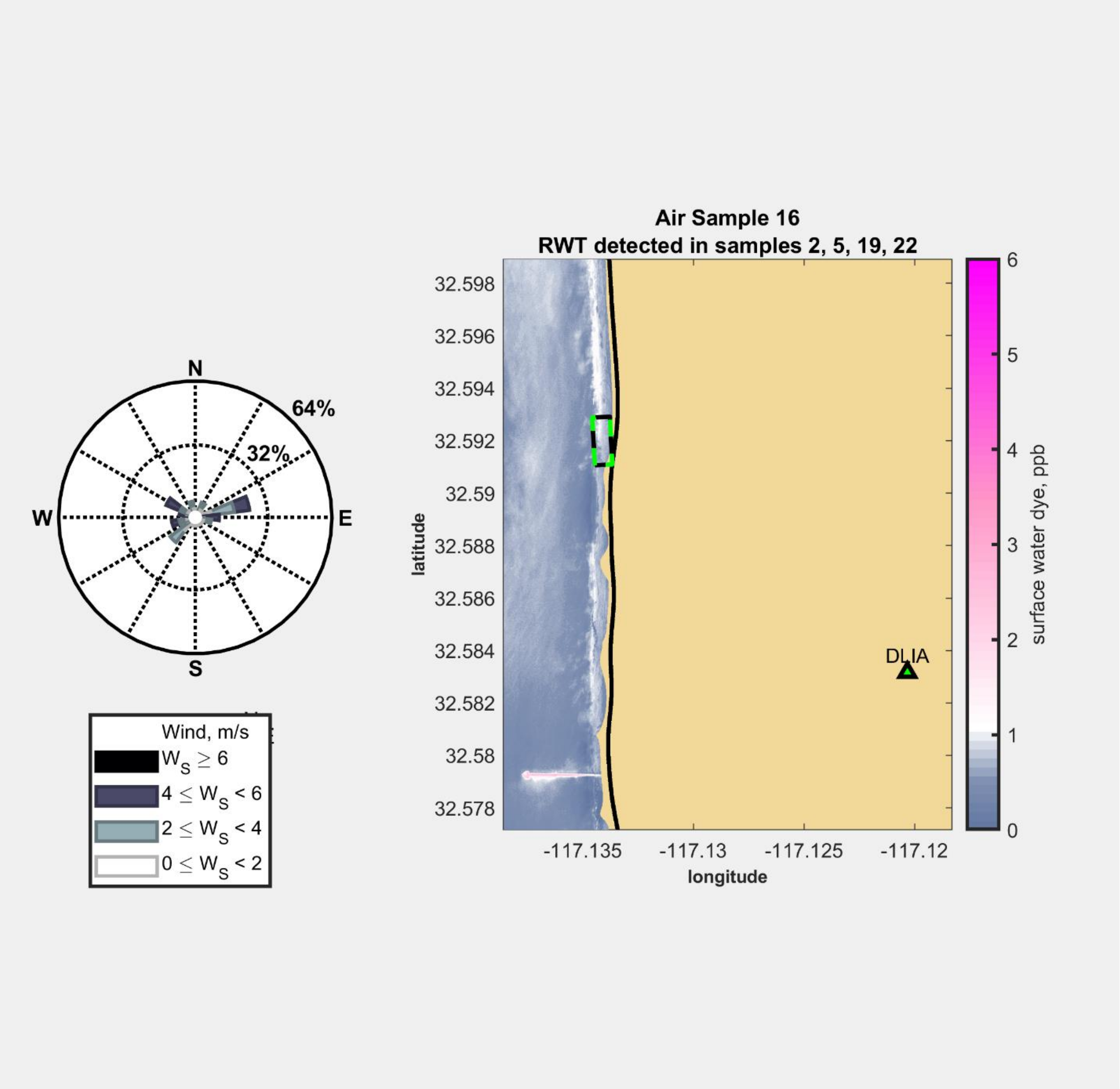

N
S
W
E
32%
64%
Wind, m/s
W_S ≥ 6
4 ≤ W_S < 6
2 ≤ W_S < 4
0 ≤ W_S < 2
Air Sample 16
RWT detected in samples 2, 5, 19, 22
latitude
longitude
32.598
32.596
32.594
32.592
32.59
32.588
32.586
32.584
32.582
32.58
32.578
-117.135
-117.13
-117.125
-117.12
DLIA
surface water dye, ppb
0
1
2
3
4
5
6

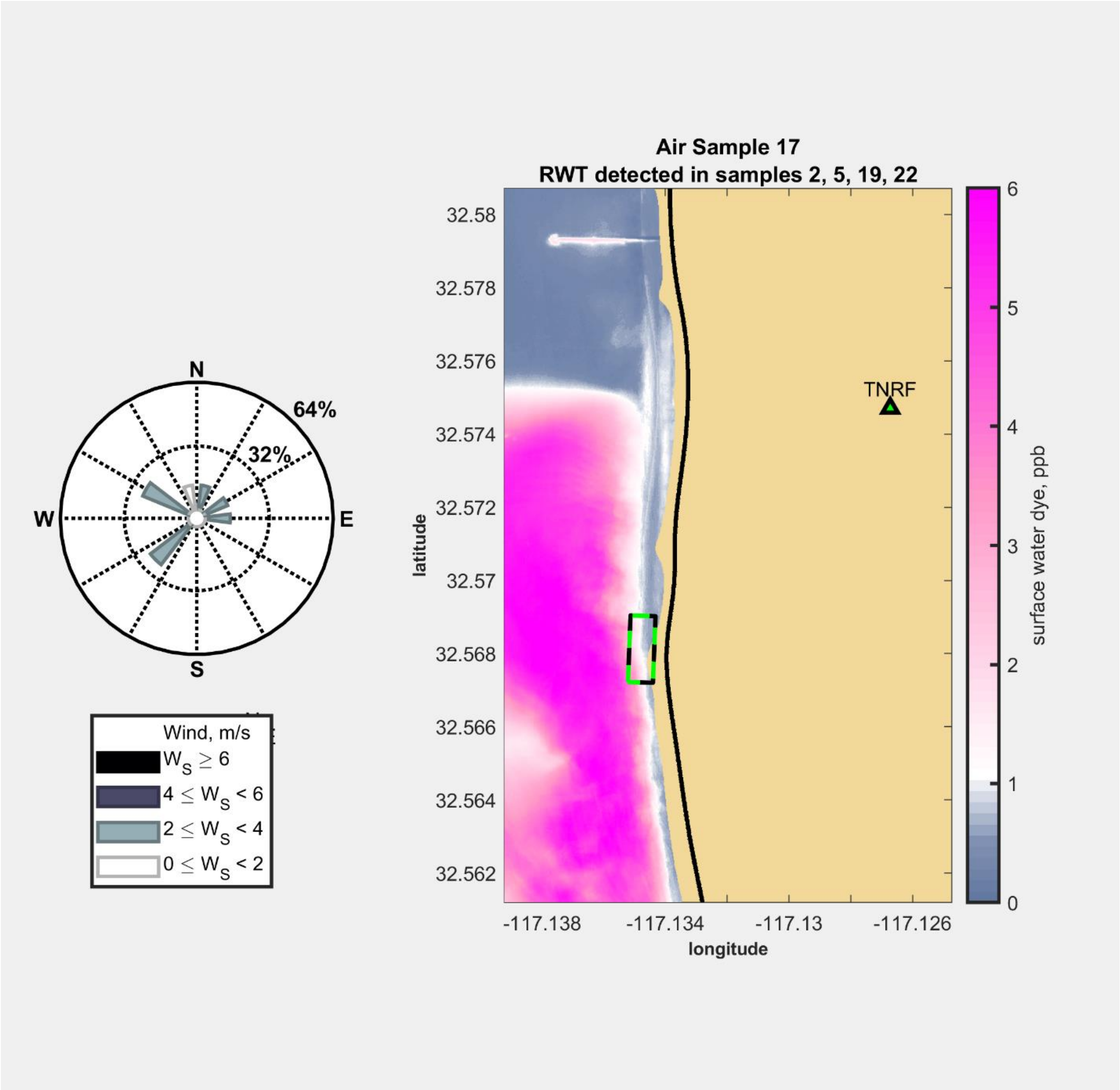

Air Sample 17
RWT detected in samples 2, 5, 19, 22
N
S
W
E
64%
32%
Wind, m/s
$W_S \geq 6$
$4 \leq W_S < 6$
$2 \leq W_S < 4$
$0 \leq W_S < 2$
TNRF
latitude
longitude
surface water dye, ppb
32.58
32.578
32.576
32.574
32.572
32.57
32.568
32.566
32.564
32.562
-117.138
-117.134
-117.13
-117.126
0
1
2
3
4
5
6

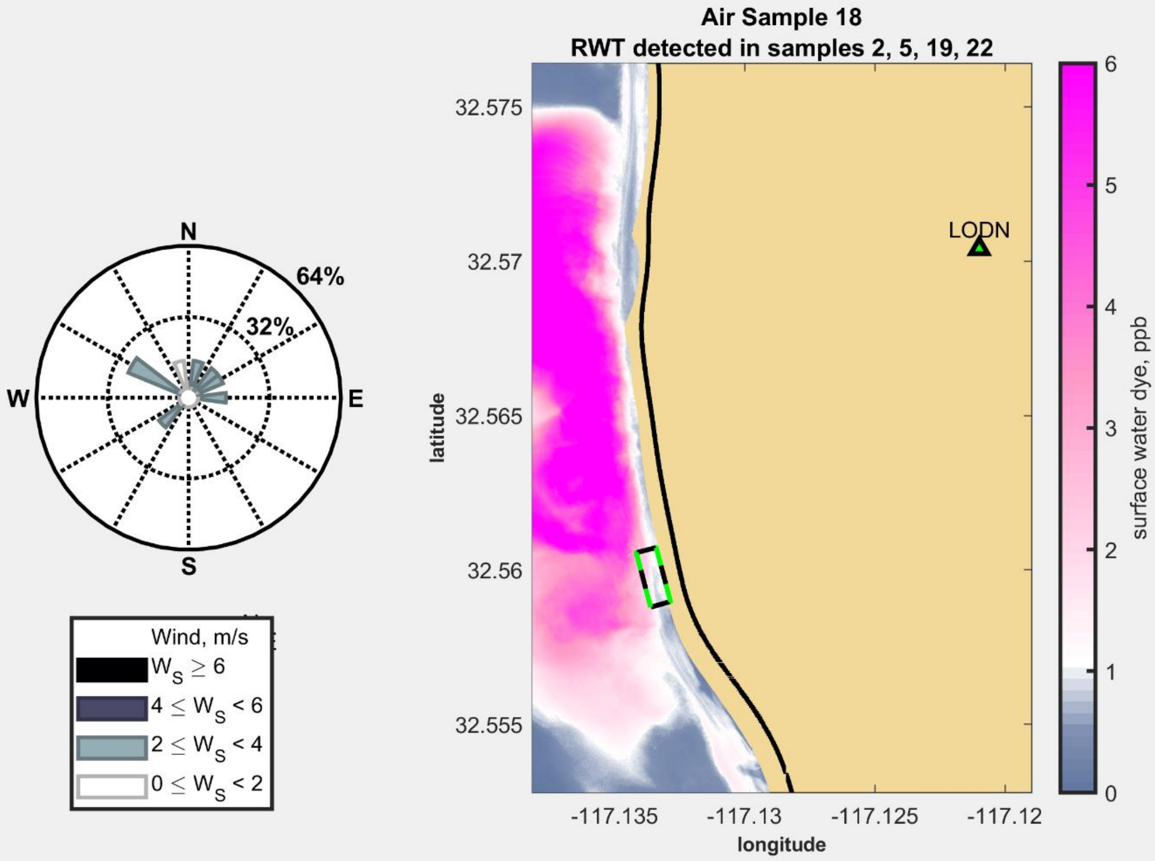

N
64%
32%
W
E
S
Wind, m/s
W_S ≥ 6
4 ≤ W_S < 6
2 ≤ W_S < 4
0 ≤ W_S < 2
Air Sample 18
RWT detected in samples 2, 5, 19, 22
LODN
latitude
longitude
surface water dye, ppb
32.575
32.57
32.565
32.56
32.555
-117.135
-117.13
-117.125
-117.12
0
1
2
3
4
5
6

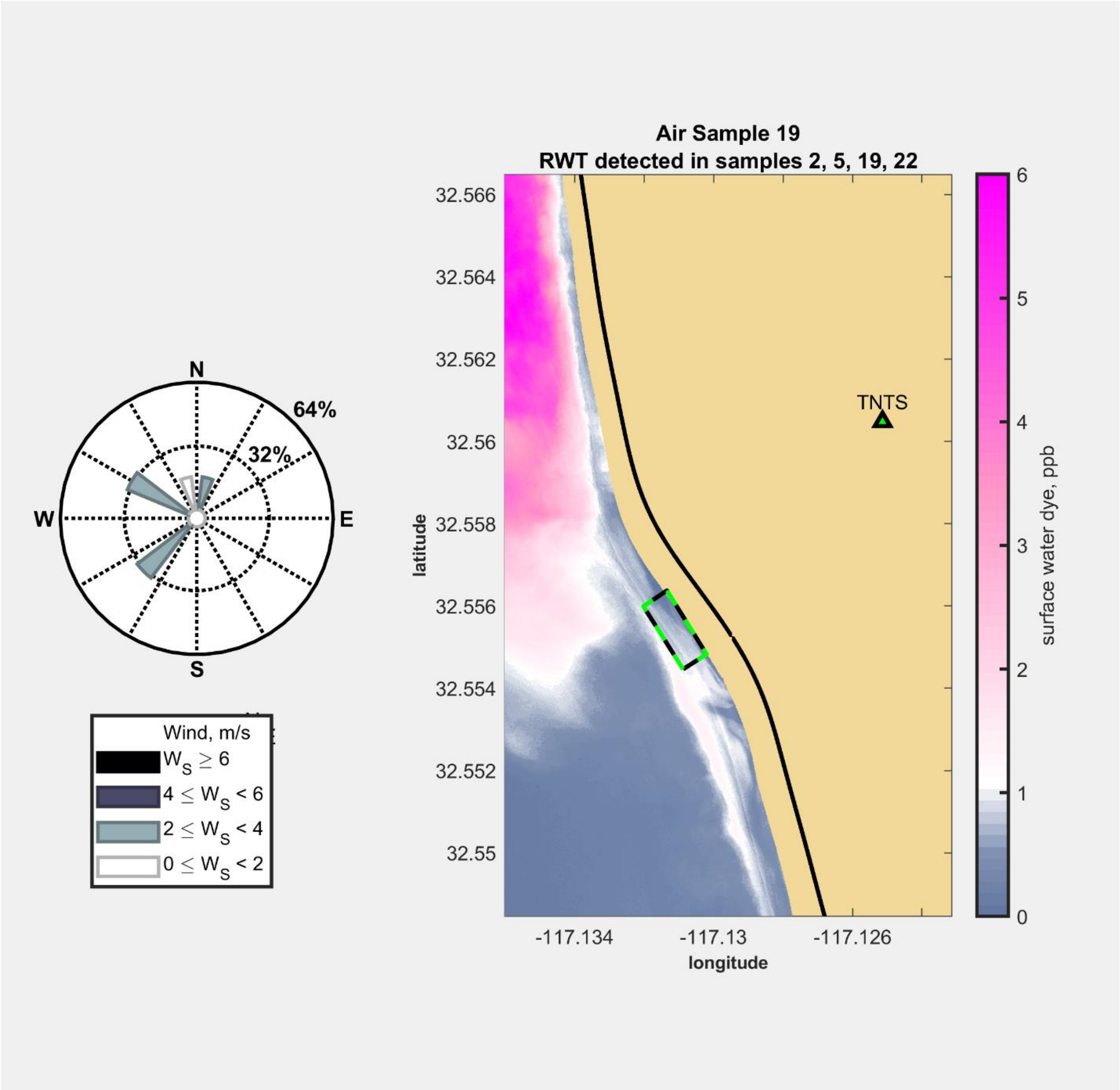

Air Sample 19
RWT detected in samples 2, 5, 19, 22
N
E
S
W
64%
32%
Wind, m/s
$W_S \geq 6$
$4 \leq W_S < 6$
$2 \leq W_S < 4$
$0 \leq W_S < 2$
latitude
longitude
32.566
32.564
32.562
32.56
32.558
32.556
32.554
32.552
32.55
-117.134
-117.13
-117.126
TNTS
surface water dye, ppb
0
1
2
3
4
5
6

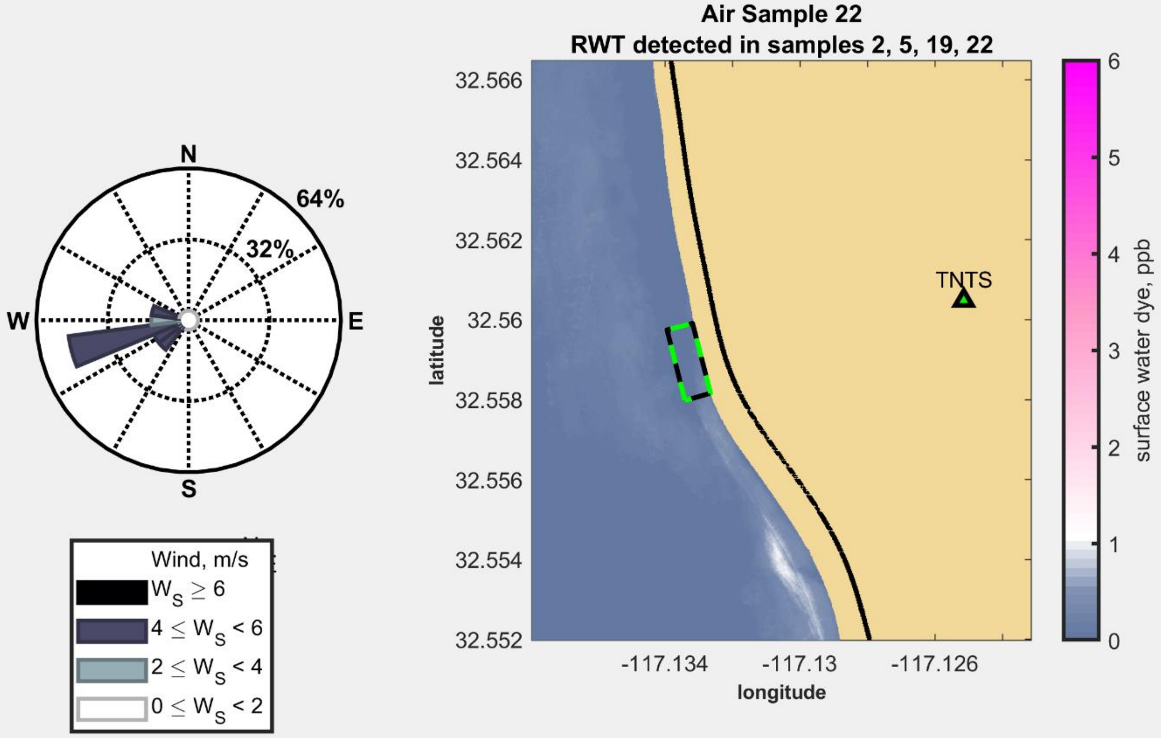

N
S
E
W
64%
32%
Wind, m/s
$W_S \geq 6$
$4 \leq W_S < 6$
$2 \leq W_S < 4$
$0 \leq W_S < 2$
Air Sample 22
RWT detected in samples 2, 5, 19, 22
TNTS
latitude
longitude
surface water dye, ppb
32.566
32.564
32.562
32.56
32.558
32.556
32.554
32.552
-117.134
-117.13
-117.126
0
1
2
3
4
5
6
