# Supplementary material for: Airborne transmission pathway for coastal water pollution
Source: PeerJ. 2021 Jun 7;9:e11358. doi: 10.7717/peerj.11358 (PMC8191489; doi:10.7717/peerj.11358)
Supplement: Supplemental Information 5 — More details on methods used [file peerj-09-11358-s005.docx]

**Airborne exposure to coastal water pollution**

Matthew A. Pendergraft^1^, Derek J. Grimes^1^, Sarah N. Giddings^1^, Falk Feddersen^1^, Charlotte M. Beall^1^, Christopher Lee^1^, Mitchell V. Santander^2^, Kimberly A. Prather^1, 2^

^1^ Scripps Institution of Oceanography, University of California San Diego, La Jolla, CA, USA

^2^ Dept. of Chemistry & Biochemistry, University of California San Diego, La Jolla, CA, USA

Corresponding Authors:

Kimberly A. Prather^1^ & Matthew A. Pendergraft^1^

9500 Gilman Drive, Mail Code 0206

La Jolla, CA 92093-0206

Email address: kprather@ucsd.edu , mpenderg@ucsd.edu

SUPPLEMENTAL MATERIALS

Supplemental Methods

**Rhodamine WT Identification and Quantification in Aerosol Samples**

Analyzing a RWT standard provided the RWT fluorescence pattern for visual detection in the samples (Fig. S2). The maximum fluorescence from the five highest calibration curve concentrations (15 total scans) occurred at a total of 3 excitation/emission wavelength pairs (555/580, 560/580, and 560/585 nm), all in the vicinity of the reported wavelengths of maximum RWT fluorescence (558/582 nm). Fluorescence quantification used the mean fluorescence intensity from these three wavelength pairs for each sample.

Four aerosol field blanks were collected Oct. 7-8, 2015, after dye from the first dye release had dissipated for 2 weeks. None of the four blanks presented the RWT fluorescence signature. They were processed identically as the aerosol samples to generate dye concentrations in air, which provided values for measurement error (Table 1). This produced a mean and standard deviation of 9.5±7.1 pg/m^3^ which was used along with eqn. (1) to generate our limit of detection of 32.8 pg/m^3^ which serves as one of two criteria for detecting dye in air samples^1^. We observed significant variability in the background fluorescence of our air samples and field blanks, as evidenced by varying fluorescence intensities between features in the excitation-emission matrices, which we attribute to atmospheric particle loads that varied across different samples. To account for this variability, a background subtraction was applied to all standards and samples to remove scatter off the 1/1 excitation/emission line and other noise, so as to not be quantified as RWT fluorescence. After reviewing all spectra, an excitation/emission region was selected which did not show strong signal in any samples or standards, but did include the 1/1 excitation/emission line (see boxes in Figure S2). For each spectrum, the mean fluorescence intensity from this region was subtracted from the entire spectrum. Then RWT fluorescence was calculated from the fluorescence intensity at the three excitation/emission pairs as described.

**Rhodamine WT concentrations in near the surfzone upwind of the aerosol samplers**

To determine the sea surface dye concentrations upwind of the aerosol samplers ([dye]_sea_ (ppb); Table 1 and x axis in Figure 3), upwind vectors were derived using wind data from a local meteorological station (KNRS, Fig. 1) and interpolated to the time intervals of aerial dye measurements. No lag between source and wind times was imposed because the observed ~5 m/s winds advected material from surfzone to sampler (~1.5 km) in ~5 min, much shorter than the multi-hour aerosol sampling intervals. The intersection of the interpolated upwind vector with the tidally varying shoreline location, estimated using the 2012 NOAA Tsunami DEM and local water-level (including tides, but neglecting wave set-up), provided the center location for the upwind dye source. A 200 m alongshore by 100 m offshore sea surface dye concentration window, relative to the upwind shoreline intersection location, was extracted for each remote sea surface dye measurement taken during each aerosol sampling period. The spatial and temporal average of the sea surface dye concentration windows provided an approximate average upwind sea surface dye concentration over each aerosol sampling period (SS dye (ppb); Table 1). This allowed us to accurately define the RWT aerosol source waters and quantitatively relate RWT concentrations in the ocean and atmosphere. This box was limited to 100 m in the offshore direction as SSA generation was mainly expected to occur in the surfzone region of depth-limited breaking waves.

**References**

1. Armbruster, D. A. & Pry, T. Limit of Blank , Limit of Detection and Limit of Quantitation. **29**, 49–52 (2008).
